# Reversible zwitterionic coordination enables rapid, high-yield, and high-purity isolation of extracellular vesicles from biofluids

**DOI:** 10.1126/sciadv.adf4568

**Published:** 2023-04-14

**Authors:** Qiang Li, Zhaowei Zhang, Fengchao Wang, Xiang Wang, Saisong Zhan, Xiaoqing Yang, Chen Xu, Dingbin Liu

**Affiliations:** ^1^ State Key Laboratory of Medicinal Chemical Biology, Research Center for Analytical Sciences, Tianjin Key Laboratory of Molecular Recognition and Biosensing, and Frontiers Science Center for New Organic Matter, College of Chemistry, Nankai University, Tianjin 300071, China.; ^2^ Tianjin Institute of Urology, The Second Hospital of Tianjin Medical University, Tianjin 300211, China.; ^3^ Department of Colorectal Surgery, Tianjin Union Medical Center, Tianjin Institute of Coloproctology, School of Medicine, Nankai University, Tianjin 300071, China.

## Abstract

Extracellular vesicles (EVs) hold great clinical value as promising diagnostic biomarkers and therapeutic agents. This field, however, is hindered by technical challenges in the isolation of EVs from biofluids for downstream purposes. We here report a rapid (<30 min) isolation method for EV extraction from diverse biofluids with yield and purity exceeding 90%. These high performances are ascribed to the reversible zwitterionic coordination between the phosphatidylcholine (PC) on EV membranes and the “PC-inverse” choline phosphate (CP) decorated on magnetic beads. By coupling this isolation method with proteomics, a set of differentially expressed proteins on the EVs were identified as potential colon cancer biomarkers. Last, we demonstrated that the EVs in various clinically relevant biofluids, such as blood serum, urine, and saliva, can also be isolated efficiently, outperforming the conventional approaches in terms of simplicity, speed, yield, and purity.

## Teaser

A reversible chemical recognition system enabled rapid isolation of extracellular vesicles with high purity and yield.

## INTRODUCTION

Extracellular vesicles (EVs) are phospholipid bilayer-enclosed structures ranging in size from 30 nm to several micrometers that carry specific proteins, nucleic acids, and small-molecule metabolites of their host cells (*1*–*4*). EVs are frequently identified in various biofluids, such as serum (*5*), urine (*6*), saliva (*7*), and tears (*8*), where they could indicate the presence, development, and therapeutic response of numerous diseases (*9*–*13*). Therefore, EVs have been recognized as one of the most promising liquid-biopsy biomarkers (*14*). Some cancer-specific proteins are enriched on EV membrane, making it feasible to capture and detect the cancer-derived EVs via corresponding antibodies or aptamers (*15*, *16*). However, the detection of EVs remains a technical challenge due to their low concentration in biofluids and high degree of heterogeneity in size and composition (*17*, *18*). Besides, the high-abundance contaminants such as nanoscale lipoproteins and cell-free nucleic acids often coexist in the biofluids to interfere with EV detection, thus reducing analytical sensitivity and accuracy (*19*, *20*). These issues can be addressed by isolating EVs from the biofluids for downstream analysis. Furthermore, as a type of naturally generated nanovesicles, EVs have been widely used as drug delivery carriers and even therapeutic agents (*21*, *22*). Their therapeutic effects are determined by the purity, integrity, and activity of EVs. At present, the research and deployment of EVs are severely impeded by the dearth of high-yield and high-purity isolation approaches.

Despite the fact that ultracentrifugation (UC) has long been regarded as the “gold standard” for EV isolation, its extremely poor yield (often less than 30%) and lengthy processing time preclude its application in clinical settings (*23*). Polyethylene glycol (PEG)–based precipitation is frequently used to enrich EVs, but it is ineffective at distinguishing EVs from the cell-free impurities in biofluids, resulting in low purity (*24*). Immunomagnetic isolation methods based on biomarker-antibody recognition have been extensively studied; they are largely limited by the vast heterogeneity of biomarker expression on the EV surfaces (*25*, *26*). For example, epithelial cell adhesion molecule (EpCAM) represents a common cancer biomarker that is used for immunomagnetic enrichment of cancer cell–derived EVs; it is only expressed in fewer than 50% of cancerous EVs (*27*). Although the past decade has witnessed several cutting-edge EV isolation methods, including microfluidics (*28*), size exclusion chromatography (*29*), multi-step ultrafiltration (*30*), and phosphatidylserine affinity (*31*), they are labor-intensive, expensive, and require specialized staff. The development of powerful EV isolation platforms that can be quickly completed with high yield and high purity, ideally ones without resorting to complicated processes and expensive instruments, is crucial for EV-based diagnostics and therapy to become clinically effective.

We here report a reversible zwitterionic coordination strategy to enable the rapid isolation of EVs from diverse biofluids, breaking through the long-standing dilemma of EV studies, i.e., harvesting EVs from real samples with high yield and purity simultaneously (*23*–*26*). This strategy is based on the specific polyvalent interactions between the phosphatidylcholine (PC) on EV membranes and the “PC-inverse” choline phosphate (CP) anchored on magnetic beads (MBs). PC is the most abundant headgroup of phospholipids, which stand as the fundamental component of EVs (*32*, *33*). Therefore, the PC-CP interactions are expected to capture all the EVs other than the non-EV counterparts (*34*, *35*). In addition, CP is a zwitterionic moiety characteristic of antifouling effect (*36*, *37*), which minimizes the nonspecific adsorption of biological contaminants on the beads. Furthermore, the PC-CP coordination can be regulated reversibly by slightly altering the ambient temperature, which allows the instantaneous release of EVs from the beads for downstream investigations (*38*–*40*). The potential applications of this innovative technique were demonstrated by coupling with proteomics for the identification of cancer biomarkers and further isolating the EVs from a variety of clinically relevant biofluids. This reversible zwitterionic coordination–based platform enables EV isolation with a notably faster processing speed, yield, and purity when compared to the gold standard UC and other conventional approaches, implying great promises for clinical translation.

### RESULTS

### CP synthesis and MB@CP optimization

**Fig. 1.**
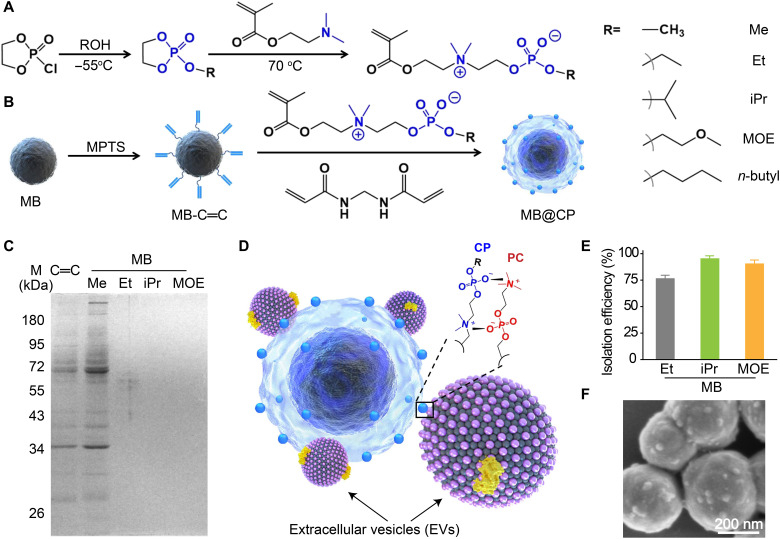
Preparation of MB@CPs and optimization of CP monomers. Schematic illustrations of the synthesis of (**A**) CP monomers and (**B**) corresponding MB@CPs. The zwitterionic CPs were installed on the MB surfaces via precipitation polymerization. (**C**) SDS–polyacrylamide gel electrophoresis (SDS-PAGE) analysis of the proteins isolated from the MB@CPs bearing different headgroups. All the MB@CPs (1 mg/ml) were incubated with 10% fetal bovine serum (FBS) before magnetic isolation of the proteins. (**D**) Mechanism for the adhesion of EVs on a MB@CP via the high-affinity CP-PC interaction, which forms two quaternary nitrogen-phosphorus pairs to constitute a quadrupole. (**E**) Isolation efficiencies of the MB@CPs bearing different headgroups such as Et (ethyl), iPr (isopropyl), and MOE (methoxyethyl). (**F**) Scanning electron microscopy image of SW620 EVs adhered on the surfaces of MB@CP-iPr. Scale bar, 200 nm.

We set out this study with the synthesis of CP monomers, which were then grafted on the MB surfaces via in situ polymerization. It took two steps to synthesize the CP monomers (Fig. 1A). First, 2-chloro-2-oxo-1,3,2-dioxaphospholane reacted with ROH at −55°C to produce 2-RO-2-oxo-1,3,2-dioxaphospholane, followed by a ring-opening nucleophilic substitution under the treatment of 2-(dimethylamino)ethyl methacrylate to produce CP monomers (*35*). For the synthesis of 2-methacryloyloxyethyl CP (CP-Me), a degradation event occurred during the ring-opening reaction, leading to the formation of by-products, as characterized by ^1^H and ^35^P nuclear magnetic resonance (NMR) spectra (figs. S1 to S3) (*41*). Lengthening the alkyl chains of the substituent R is an efficient way to boost the chemical stability of the CP monomers (*42*, *43*). Therefore, we prepared a set of CP derivatives bearing ethyl- (Et-), isopropyl- (iPr-), methoxyethyl- (MOE-), and *n*-butyl. Taking CP-iPr as an example, the ^1^H NMR, ^35^P NMR, and mass spectroscopy (MS) spectra indicated that nearly no by-products were generated during the reaction process (figs. S4 to S6). The ^1^H NMR and MS characterizations of Et-, MOE-, and *n*-butyl–bearing CP monomers are shown in figs. S7 to S12.

Next, we prepared MBs via a hydrothermal method (Fig. 1B) (*44*). The as-prepared MBs are mono-dispersed suspensions with a mean hydrodynamic diameter of 459 nm (fig. S13). The MB surfaces were first modified with 3-methacryloxypropyltrimethoxy silane to produce a silica shell immobilizing with C═C bonds. The C═C-covered MBs (MB-C═C) were then reacted with the CP monomers in the presence of C═C-conjugated crosslinkers, resulting in MB@CP core-shell nanogel structures wherein the exposed CP groups are designed to recognize the PC on EVs and simultaneously resist nonspecific protein adsorption. Consequently, the particle size of all the five MB@CPs increased, as recorded by the dynamic light scattering (DLS) data (fig. S13). It should be noted that the average hydrodynamic diameter of CP–*n*-butyl–modified MBs (MB@CP-*n*-butyl) increased to approximately 1 μm with a wide distribution, implying particle aggregation. A likely reason for this phenomenon might be ascribed to the high hydrophobicity of the *n*-butyl head. Poor dispersion of MB@CP-*n*-butyl in aqueous solutions limits its applications.

Several analytical methods were used to characterize the as-prepared MB@CPs. Because all five MB@CPs were prepared with the same chemistry, we here only took MB@CP-iPr as an example to illustrate the characterizations. Upon the formation of MB@CP-iPr, two bands at 1725 and 1234 cm^−1^ (fig. S14) were observed in the Fourier transform infrared spectrum, which were attributed to the P─O bonds in the CP monomers and N─H bonds in the crosslinkers, respectively. The well-defined core-shell nanostructures were directly characterized with transmission electron microscopy (TEM; fig. S15). We further performed energy-dispersive x-ray spectroscopy analysis to evaluate the composition of MB@CP-iPr. The elemental signals of Fe, Si, and P correspond to the MB core, SiO_2_ shell, and CP gel, respectively (fig. S16). The thermogravimetric analysis profile of MB@CP-iPr had a pronounced step at approximately 650°C (fig. S17), which was attributed to the weight loss of the CP-constituted nanogel structure from the MB surfaces. Last, the magnetic hysteresis loop (fig. S18) and x-ray diffraction (fig. S19) results indicated that the CP nanogel encapsulation would scarcely influence the superparamagnetic property of MBs.

The CP-modified nanomaterials should have a neutral net charge surface and show excellent antifouling ability because of their zwitterionic property. However, the zeta potential of MB@CP-Me was measured to be +31 mV (fig. S20), supporting the above observation that CP-Me tends to degrade spontaneously into the positively charged quaternary ammonium. The subsequent anti-protein adsorption experiment showed that MB@CP-Me captured many more proteins than the CP-Et–, CP-iPr–, and CP-MOE–modified MBs (Fig. 1C), further confirming the transformation of CP-Me into quaternary ammonium.

We then evaluated the isolation efficiencies of the CP-Et–, CP-iPr–, and CP-MOE–modified MBs by incubating them with DiO-labeled EVs, as illustrated in Fig. 1D. Through simply comparing the fluorescence intensity of DiO in the supernatants before and after removing the EVs with MB@CPs, the isolation efficiencies of CP-Et–, CP-iPr–, and CP-MOE–modified MBs were estimated to be 78, 95, and 90%, respectively (Fig. 1E). This result implies that the CP-iPr–modified surfaces show the highest adhesion force than those modified with CP-Et or CP-MOE to interact with PC-modified substrates (*43*). Therefore, we chose MB@CP-iPr for the following experiments.

### Optimization of EV isolation conditions using MB@CP-iPr

To optimize the isolation conditions and further evaluate the performance of MB@CP-iPr for EV isolation, model EVs were prepared by UC from the fetal bovine serum (FBS)–free cell culture medium supernatants of SW620 cells (*25*). Note that the model EVs only included small EVs because the large vesicles were removed by 10 K centrifugation. The model EVs were characterized by cryo–electron microscopy (cryo-EM) and nanoparticle tracking analysis (NTA) as guided by MISEV2018 (*45*). They exhibited a characteristic saucer-shaped morphology under cryo-EM (fig. S21), with a size distribution from 30 to 400 nm (fig. S22). The EV pellets were carefully redispersed into the FBS-free cell culture medium, where the EV concentration was measured to be 10 μg/ml (~10^9^ EVs) by the bicinchoninic acid (BCA) protein quantification assay.

With the model EVs in hands, we first evaluated the EV capture efficiency of MB@CP-iPr at different concentrations. The capture efficiency rose gradually as the increase of bead concentrations; the highest efficiency reached 96% with MB@CP-iPr (1 mg/ml) (fig. S23). Further increase of the MB@CP-iPr concentration did not improve the efficiency. Besides, we found that it took only 5 min to achieve high-efficiency enrichment of EVs (fig. S24). The scanning electron microscopy image confirmed the efficient capture of EVs on the surfaces of MB@CP-iPr (Fig. 1F). Considering that the concentrations of EVs in different biofluids vary substantially, we tested the isolation efficiency of MB@CP-iPr for EVs at a variety of concentrations. The MB@CP-iPr afforded isolation efficiencies higher than 90% (fig. S25) at the EV concentrations ranging from 0.5 to 10 μg/ml, a wide distribution in common human biofluids.

**Fig. 2.**
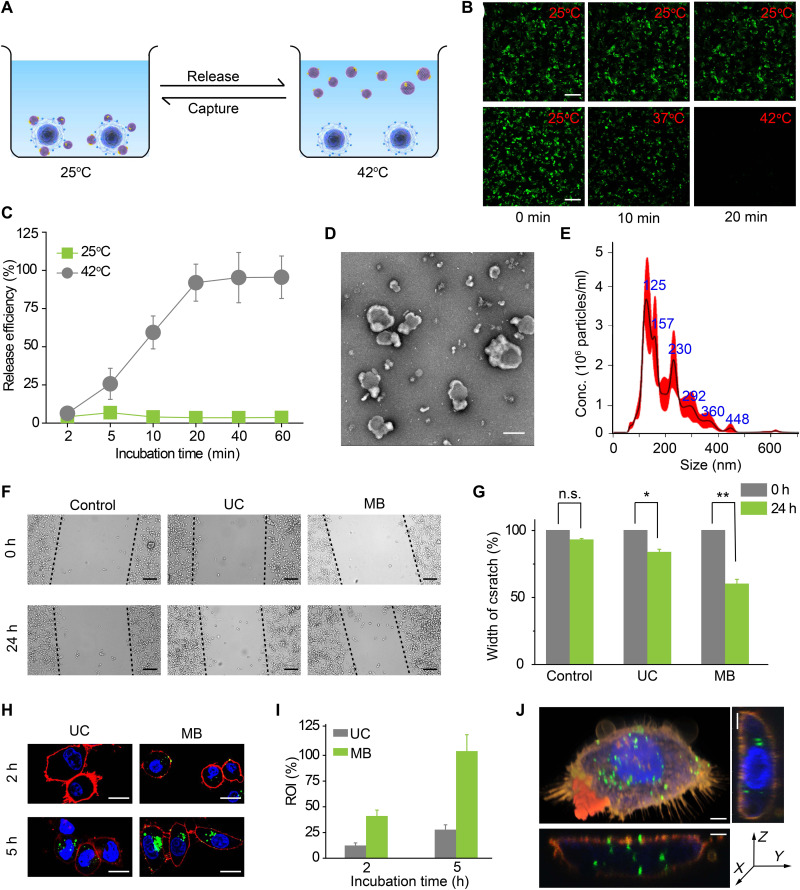
Temperature-mediated release of EVs from MB (denote as MB@CP-iPr here). (**A**) Schematic illustration of the temperature-responsive release of SW620 EVs from MB@CP-iPr. (**B**) Fluorescence images showing the release of EVs from MB@CP-iPr after temperature increase from 25°C to 42°C. Scale bar, 20 μm. (**C**) The time-dependent release efficiencies of EVs from MB@CP-iPr in (B). Error bars denote mean ± SEM (*n* = 3). (**D**) Typical morphology of the EVs characterized by cryo-EM image (scale bar, 100 nm) and (**E**) size distribution of the EVs measured by NTA. (**F**) Wound healing assay of SW480 cells incubated with SW620 EVs isolated by UC and MB, respectively. Migration was assessed at 24-hour time point after wounding. The absence of SW620 EVs was set as control. Scale bars, 50 μm. (**G**) Width of the scratches from the different groups in (F). Error bars denote mean ± SEM (*n* = 3; n.s., not significant; *P* > 0.05, *P < 0.05, and **P < 0.01; two-tailed *t* test.). (**H**) Confocal images of SW480 cells after incubating with SW620 EVs isolated by UC and MB for 2 and 5 hours, respectively. Blue, Hoechst 33343; green, EVs-DiO); red, DiI. Scale bars, 10 μm. (**I**) Cell uptake efficiencies were determined by calculating the region of interest (ROI) of the green signals with ImageJ software. The ROI of MB group at 5 hours was set at 100%. Error bars denote mean ± SEM (*n* = 3). (**J**) 3D confocal images of a SW480 cell after incubating with SW620 EVs isolated by MB. Blue, Hoechst 33343; green, EVs-DiO; and red, DiI). Scale bars, 2 μm. h, hours.

More impressively, the captured EVs on MB@CP-iPr could be released by increasing the ambient temperature to break down the CP-PC interactions (Fig. 2A). The DiO-labeled EVs that had been captured by MB@CP-iPr were used for the temperature-induced EV release testing. Distinct green fluorescent signals were observed for the MB@CP-iPr adsorbed by the DiO-labeled EVs. Once increasing the ambient temperature, the fluorescence signals on the MB@CP-iPr weakened gradually (Fig. 2B). When the temperature reached 42°C, no noticeable fluorescence signals were observed on the beads. By contrast, the fluorescence intensity on the same beads loaded with DiO-labeled EVs did not change when they were incubated at 25°C. The results indicated that the captured EVs on the MB@CP-iPr can be released by simply changing the incubation temperature, without resorting to the addition of any reagents. The subsequent release kinetics experiments demonstrated that approximately 92% of EVs were released at 42°C after 20 min of incubation (Fig. 2C). The cryo-EM and NTA results showed that the EVs released from the MB@CP-iPr remained classical vesicle shape with excellent monodispersity (Fig. 2, D and E).

Because the temperature-dependent release process did not require any additional reagents or complicated multi-step operations, the released EVs might maintain native biological activity and functions. SW480 and SW620 cell lines were used for testing the EV activity. These two cell lines originated from the same patient with colon cancer with different levels of metastasis; SW480 was derived from primary colon cancer, while SW620 was derived from metastatic colon cancer (*46*). A solution (3 ml) of model EVs derived from the highly aggressive SW620 cells was divided into two parts, which were respectively isolated by MB@CP-iPr and UC (as a comparison) and resuspended in 1.5 ml of FBS-free medium. The two types of resuspended EV media were incubated with the low-invasive SW480 cells in a wound healing assay. The results showed that, after incubation with the MB@CP-iPr–released EVs, the SW480 cells had a 40% wound closure rate (Fig. 2, F and G), which was higher than that achieved with the UC group (20%). To figure out the reason why the MB@CP-iPr–released EVs showed a higher wound closure rate than the UC-collected EVs, the EV concentrations in the two groups were made consistent by a BCA protein quantification assay. As shown in fig. S26, the wound closure rates are positively correlated with the EV concentrations in both the MB@CP-iPr and UC groups. However, there was no statistical difference between the two groups. We note that when the EV concentrations reached 10 μg/ml, the MB@CP-iPr–released EVs showed higher activity than the UC-isolated EVs to prompt cell migration. These findings showed that the primary factor affecting the rate of wound closure is EV concentration, with EV integrity playing a supporting role.

Cellular uptake experiments were conducted to further identify the biological activity of EVs isolated by the two methods. The EVs isolated by UC or MB@CP-iPr from 3 ml of the model EV solution were first labeled with DiO and then incubated with SW480 cells for different periods. As shown in Fig. 2 (H and I), the fluorescence signals from the MB@CP-iPr group were 2.6 times stronger than those from the UC group at 5 hours. Three-dimensional (3D) fluorescence confocal imaging showed that the EVs were uniformly distributed in the cytoplasm (Fig. 2J). This result suggests that the temperature-induced EV release could be more effective than UC in preserving the biological activity of EVs.

To verify the influence of the two isolation methods on the integrity of EVs, the total RNA contents extracted from UC- or MB@CP-iPr–isolated SW620 EVs were tested by agarose gel electrophoresis (fig. S27). RNAs in EVs are considered an essential factor influencing the biological function of target cells. The results showed that the EVs isolated by MB@CP-iPr had a higher RNA concentration than those isolated by UC. We reasoned that the EV pellets obtained by UC may rupture to release the internal RNAs during the redispersion operation.

Considering the excellent stability of the CP-gel structure formed by covalent bonds, the capture efficiencies of MB@CP-iPr were tested for five cycles by the Western blotting assay with cluster of differentiation molecule 9 (CD9; a universal EV marker) (*47*). The results indicate that MB@CP-iPr could be recycled for EV isolation (fig. S28), thereby substantially reducing the cost.

### Determination of EV isolation yield and purity by MB@CP-iPr

To determine the isolation efficiency of MB@CP-iPr for EV capture in a complex environment, artificial biofluid samples were prepared by mixing the model SW620 EVs with different concentrations of EV-free FBS (*22*). The EV-free FBS contains various molecules such as growth factors, proteins, vitamins, trace elements, hormones, etc. (*48*), which are frequently used to evaluate the effect of complex environments on the yield and purity of EV isolation.

**Fig. 3.**
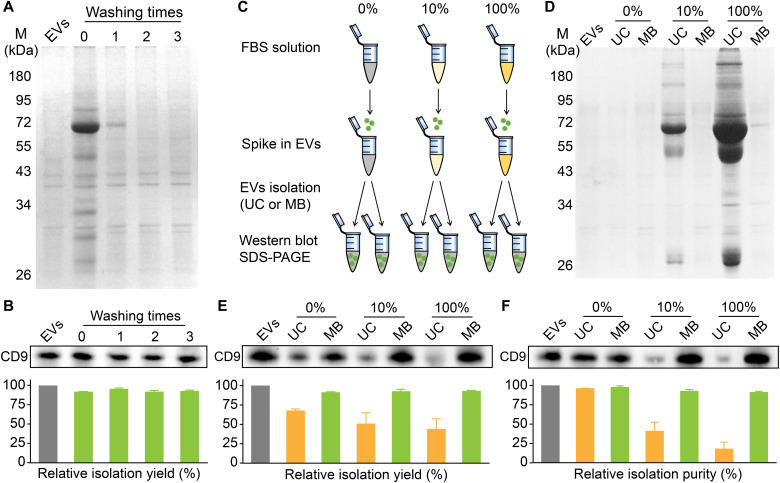
Comparison of the yield and purity of EVs isolated from complex solutions by UC and MB (denote as MB@CP-iPr here). (**A**) SDS-PAGE analysis of the protein contamination and (**B**) Western blotting analysis of the EV marker (CD9). M denotes protein marker; EVs denote the model EVs sample (10 μg/ml, ~10^9^ EVs); “washing time 0” denotes the mixture of the model EVs released from the EV-adsorbed MBs and 10% FBS without PBS washing, while “washing times 1, 2, 3” denote the same EVs released from the EV-adsorbed MBs after washing with PBS for different times. (**C**) Schematic diagram of the experiment design. (**D**) SDS-PAGE analysis of protein components in different FBS concentration groups (0, 10, and 100%) where the EVs were isolated by UC and MB, respectively. (**E**) Equal-sample-volume (10 μl of EV samples, left) and (**F**) equal-protein-amount (5 μg, right) Western blotting analysis of CD9 in samples at different FBS concentrations where the EVs were isolated by UC and MB, respectively. The relative band intensity of CD9 is presented in the bar charts (*n* = 3 independent experiments).

Owing to the excellent antifouling effect and high EV capture efficiency of MB@CP-iPr, nearly all the protein impurities were removed from the beads after washing with phosphate-buffered saline (PBS) for multiple times while the EVs adsorbed on the beads robustly (Fig. 3A). More than 90% of EVs can be isolated from the model EVs (10 μg/ml, ~10^9^ EVs) under 10% FBS conditions (Fig. 3B). To compare the performance of MB@CP-iPr and UC in the artificial biofluid samples, the model EVs were redispersed into PBS, 10% FBS spiking in PBS, and 100% FBS solutions at an identical EV concentration of ~10^9^ particles/ml. Each group was divided into two replicates (Fig. 3C). We used SDS–polyacrylamide gel electrophoresis (SDS-PAGE) to compare the protein compositions enriched by the two isolation methods. As shown in Fig. 3D, the bands for both the model EVs and those enriched from PBS (in the absence of FBS) by either UC or MB@CP-iPr are rather weak, which is due to the relatively small amounts of EV proteins. When the FBS concentrations in the artificial biofluid samples were raised, the protein contents (mainly from FBS) extracted by UC increased markedly. In contrast, the protein contents extracted by MB@CP-iPr at different FBS doses were almost identical to those of the model EV solution, indicating that no FBS proteins were enriched by MB@CP-iPr.

We further compared the isolation yield and EV purity achieved by the two methods. CD9 was analyzed by Western blotting to report the presence of EVs. Equal-sample-volume and equal-protein-concentration analyses were used to characterize the isolation yield and purity (*28*). As shown in Fig. 3 (E and F), the purity of EVs calculated through UC isolation decreased rapidly upon increasing the FBS concentration. In addition, the UC isolation would induce a loss of 40% EVs. In contrast, the MB@CP-iPr can achieve more than 90% purity and yield in isolating the EVs from the artificial biofluids even under 100% FBS conditions.

In addition to Western blotting, NTA was used to analyze the concentrations and size distributions of the EVs spiking in different concentrations of FBS. As shown in fig. S29, the particle number in the MB@CP-iPr group did not change markedly upon increasing FBS concentration, indicating that the non-EV particles (such as lipoproteins) in FBS had not been captured by MB@CP-iPr. In contrast, the particle number in the UC group was proportional to the concentration of FBS, implying that the non-EV particles had also been enriched by UC. In the MB@CP-iPr group, the particle size distribution in 100% FBS was similar to that in the absence of FBS; in the UC group, a strong peak appeared at 40 nm (fig. S30), which was mainly contributed by the lipoproteins in FBS. Through comparing the particle numbers in the absence and 100% of FBS, 91.2 ± 0.6% of the particles were assigned to EVs in the MB@CP-iPr group. In comparison, EVs contributed only 23.1 ± 4.5% of the particles in the UC group. Note that the size of many serum contaminants (such as various proteins) is less than 10 nm, which is already below the detection limit of NTA. Therefore, we used a BCA assay to further measure the protein concentrations of the EV solutions. The protein concentrations of EVs isolated by MB@CP-iPr in different FBS environments were similar to the model EVs (fig. S31), which indicated that MB@CP-iPr could achieve extremely high purity and yield in isolating the EVs from the artificial biofluids. In contrast, the levels of contamination in the UC group increased notably with the rise of FBS concentration. In the 100% FBS group, most of the proteins obtained by UC were contaminants. These results confirmed that MB@CP-iPr is advantageous over UC in terms of isolation yield and purity.

### Proteomic profiling of the isolated EVs

**Fig. 4.**
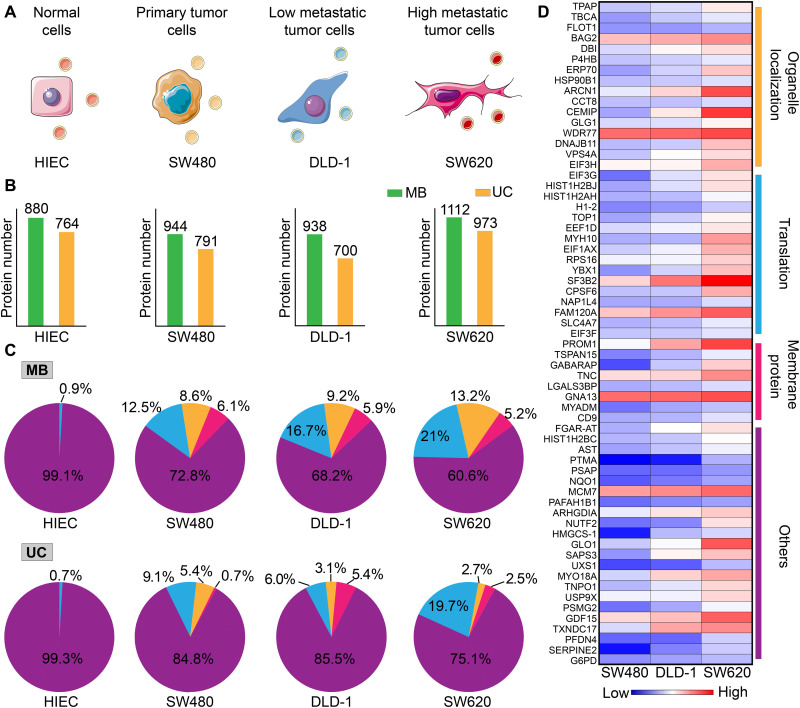
Proteomic analysis of the EVs secreted by four colon cancer–associated cell lines. (**A**) Schematic of the four cell lines with different levels of invasiveness. (**B**) The number of proteins detected in the EVs that were isolated by UC and MB (denote as MB@CP-iPr here), respectively. (**C**) Distribution reads of the EV protein-involved biological processes. Blue, RNA translation; orange, organelle localization; red, membrane protein; and purple, others. The EVs derived from the four cell lines were isolated by UC and MB, respectively. (**D**) Heatmap of the protein expressions between the EVs derived from SW480, DLD-1, and SW620 with different levels of invasiveness. Each expression difference in the heatmap is colored from blue to red to indicate low to high up-regulation.

The proteins enriched on EV surfaces, such as programmed death-ligand 1, endothelial growth factor receptor, and human epidermal growth factor receptor 2 (HER-2), are essential for EVs to perform their functions (*49*–*51*). Hence, proteomic profiling of EVs from specific cells is vital to identify protein markers of disease (*52*). The above-mentioned results have proven that MB@CP-iPr has much higher EV isolation efficiency than UC. We wanted to identify whether the higher isolation efficiency would help us in obtaining more biological information about EVs. First, we compared the protein components of EVs isolated by UC and MB@CP-iPr from a normal human intestinal epithelial cell line (HIEC) and three colon cancer cell lines (SW480, DLD-1, and SW620). SW480 is a primary cancer cell line, whereas DLD-1 and SW620 are invasive cell lines with low and high metastasis, respectively (Fig. 4A) (*53*). We collected the FBS-free culture supernatants of the four cell lines. Each supernatant was divided into two equal portions, where the EVs were isolated using the two methods. The marker proteins of EVs (Alix, Tsg-101, CD9, and CD63) were identified by Western blotting (fig. S32) (*47*), and the size distributions of EVs were identified by NTA (fig. S33). These results indicated that both UC and MB@CP-iPr could successfully isolate the EVs secreted by the four cell lines, and, undoubtedly, the isolation efficiency of MB@CP-iPr was much higher than that of UC.

The EV proteins from the four cell lines were analyzed by label-free quantitative proteomics, and their function and quantity were determined by Gene Ontology enrichment (*52*). The total amount of EV-peptide fragments isolated by MB@CP-iPr was higher than that isolated by UC (Fig. 4B). Further analysis of the proteomic information of EVs isolated by MB@CP-iPr (Fig. 4C, top) showed that the protein expression levels of RNA translation and organelle localization in EVs were substantially up-regulated with the invasiveness increase of the tumor cell lines. In particular, among the 63 identified proteins that are linked to the increase of tumor malignancy, 17 proteins (27%) and 15 proteins (23.8%) are associated with RNA translation and organelle localization, respectively (Fig. 4D). It is accepted that RNA translating proteins take part in the transport of specific RNAs into EVs to prompt tumor metastasis (*54*), while organelle localization proteins involve in the intracellular transport of EVs to facilitate cellular communication and thus prompt tumor metastasis (*55*, *56*). In contrast, despite that the same protein types can be isolated by UC (Fig. 4C, bottom), their levels are not positively associated with tumor metastasis, most likely owing to the loss of EVs caused by the low enrichment yield of UC.

Next, we calculated the differentially expressed proteins in the three types of tumor cells. To filter the data according to the criteria of *P* value < 0.05 and a fold change of >1.5, volcano plots were plotted for 21 up-regulated proteins for SW480, 16 up-regulated proteins for DLD-1, and 25 up-regulated proteins for SW620. These proteins include those closely related to tumor invasion (E-cadherin, tenascin, and cell migration–inducing and hyaluronan-binding proteins) and those assisted RNA transport (RNA binding protein and ribosomal protein) into EVs (fig. S34). Some of the EV proteins—such as growth differentiation factor 15 (GDF15) in the transforming growth factor–β superfamily and eukaryotic translation initiation factor 3 subunit G (EIF3G), eukaryotic translation elongation factor 1 delta (EEF1D), eukaryotic translation initiation factor 1A, X-chromosomal (EIF1AX), and eukaryotic translation initiation factor 3 subunit F (EIF3F) in the EIF family—are identified as potential colon cancer biomarkers. These results indicate that MB@CP-iPr is highly promising for identifying new cancer biomarkers through combining with omics.

### High-purity and high-yield isolation of EVs by MB@CP from different body fluids

Tumor-derived EVs have been explored as potential biomarkers for liquid biopsies in cancer patients (*16*). These EVs have been frequently found in cancer patient’s serum (such as breast cancer and liver cancer) (*57*, *58*), urine (such as kidney cancer and bladder cancer) (*28*), and saliva (such as nasopharynx cancer and oral cancer) (*7*). Considering the low concentration of EVs and the interference of a large number of soluble proteins in body fluids, it is necessary to develop a high-yield and high-purity isolation method for extracting EVs from different body fluids for biomarker identification and cancer diagnostics. Note that we did not remove the large vesicles from the body fluids to avoid the possible loss of molecular information about EVs. The results of NTA showed that there were no large cell fragments in the three body fluids after low-speed centrifugation, and most of the EVs were less than 400 nm in size (fig. S35).

**Fig. 5.**
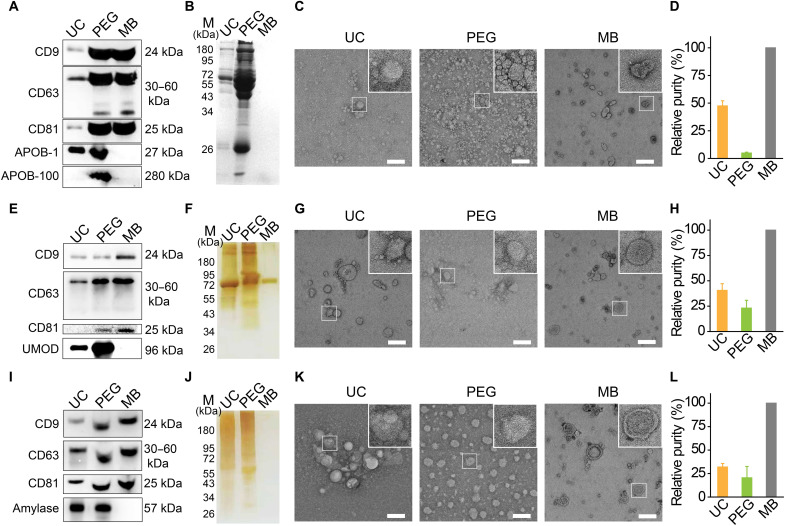
Comparison of MB (denote as MB@CP-iPr here) and other methods for isolating EVs from various body fluids. (**A**) Equal-sample-volume (2 μl of EV sample isolated from 300 μl of serum) Western blotting analysis of three common EV proteins (CD9, CD63, and CD81) in the same serum samples prepared by UC, PEG, and MB, respectively. APOB-1 and APOB-100 represent the most substantial contaminant reference of serum-EV’s purity. (**B**) SDS-PAGE analysis of protein components, (**C**) TEM images, and (**D**) relative purity of EVs isolated from the same human serum samples using the three different isolation methods. (**E**) Equal-sample-volume (5 μl of EV sample isolated from 10 ml of urine) Western blotting analysis of CD9, CD63, and CD81 in the same urine samples prepared by UC, PEG, and MB, respectively. UMOD is the most substantial contaminant reference of urine-EV’s purity. (**F**) SDS-PAGE analysis of protein components, (**G**) TEM images, and (**H**) relative purity of EVs isolated from the same human urine sample using the three different isolation methods. (**I**) Equal-sample-volume (5 μl of EVs sample isolated from 1 ml of saliva) Western blotting analysis of CD9, CD63, and CD81 in the same saliva samples prepared by UC, PEG, and MB, respectively. Amylase is the most substantial contaminant reference of saliva-EV’s purity. (**J**) SDS-PAGE analysis of protein components, (**K**) TEM images, and (**L**) relative purity of EVs isolated from the same human saliva sample using the three different isolation methods. Scale bars, 200 nm. The relative purity is presented in the bar charts (*n* = 3 independent experiments) where the enrichment purity of MB is set at 100%.

To compare the isolation yield and EV purity achieved by MB@CP-iPr with the mainstream EV isolation approaches (typically UC and PEG precipitation), we performed Western blotting to identify the presence of EV membrane proteins, including CD9, CD63, and CD81. We selected APOB-1 (a low-density lipoprotein) and APOB-100 (a very-low-density lipoprotein), the major protein contaminants of serum, to verify the purity of the obtained EVs (*28*). As shown in Fig. 5A, MB@CP-iPr shows a high yield in harvesting the EVs from human serum with high purity, as indicated by the intense bands of CD9, CD63, and CD81 and the blank bands of APOB-1 and APOB-100. By contrast, UC and PEG precipitation suffer respectively from low-yield and low-purity isolation of EVs from serum. SDS-PAGE with Coomassie blue staining further confirmed that negligible protein contaminants were enriched by MB@CP-iPr, while different levels of serum proteins were enriched by UC and PEG precipitation (Fig. 5B). Consistent with these results, the cryo-EM images show that many lipoprotein particles (~10 to 50 nm) are accompanied with the EVs isolated by UC and PEG methods, while only EVs but no lipoprotein particles were observed in the MB@CP-iPr group (Fig. 5C).

To further study the universality of MB@CP-iPr, we isolated EVs from human urine and saliva. Compared with the UC and PEG methods, the EVs purified by MB@CP-iPr showed a higher level of EV membrane proteins (CD9, CD63, and CD81) but negligible band intensity of contaminants uromodulin (UMOD) (the most abundant protein in human urine) or amylase (the primary enzyme in saliva) (Fig. 5, E and I) (*28*). SDS-PAGE with silver staining further confirmed that MB@CP-iPr can substantially minimize protein contamination to achieve high-purity isolation of EVs (Fig. 5, F and J). The cryo-EM results indicated that the EVs released from MB@CP-iPr had a complete form and clean background without protein contamination fouling (Fig. 5, G and K), greatly outperforming the other two conventional isolation methods.

The relative purity of the isolated EVs were identified by different methods. This study was first performed by dividing the amount of EVs by that of total enriched proteins (*23*). To do this, the EVs were quantified by the signal intensity of the Western blotting bands of CD9 shown in Fig. 5 (A, E, and I), while the total enriched proteins (including EVs and the coexisting protein contaminants) were determined by BCA. The mean gray values (MGVs) of the Western blotting bands of CD9 and the concentrations of total proteins isolated by UC, PEG, and MB@CP-iPr were shown in fig. S36. On the basis of these data, the enrichment purity of EVs was calculated by relating MGV to the corresponding total protein. As a consequence, the relative purities of the EVs isolated from serum by UC and PEG were determined to be 47.47 ± 4.67% and 4.86 ± 0.79%, respectively, in comparison to the enrichment purity of EVs by MB@CP-iPr that was set at 100% (Fig. 5D). Similar results were observed for the EVs isolated from the urine and saliva samples (Fig. 5, H and L).

The purity of EVs was positively associated with the ratio of the particle count to the protein amount (*45*), which was measured by NTA and BCA, respectively. Because of the specific CP-PC interactions and efficient anti-protein adsorption effect, MB@CP-iPr was able to isolate EVs from the three kinds of biofluids with much higher purity than UC or PEG (fig. S37). Furthermore, the Western blotting bands of APOB-1, UMOD, and amylase (shown in Fig. 5, A, E, and I), which stand for the most substantially protein impurities in serum, urine, and saliva, respectively, were quantified to determine the levels of protein contaminants coexisting with EVs. Considering that PEG is known to enrich a large number of contaminants during the isolation of EVs, we used the MGVs in the PEG group as a reference to estimate the relative contamination levels in the other methods. As shown in fig. S38A, the contamination levels (APOB-1) in the UC and MB groups were respectively calculated to be 53.7 and 11.9% relative to the PEG group (100%), which confirmed that MB could isolate EVs from serum with ultrahigh purity. For the other two biofluids, the achieved EV purity also follows the tendency: MB > UC > PEG, in line with the above particle-to-protein ratio results (fig. S38, B and C).

After demonstrating the high performance of MB@CP-iPr for harvesting EVs from the cell culture medium and real samples, we wanted to investigate whether the newly identified EV markers in the colon cancer cell lines could be used for the liquid biopsy of patients with colorectal cancer (CRC). CD9 and GDF15 were respectively selected as the typical EV biomarker and the potential CRC biomarker as newly identified above for the following investigations. Enzyme-linked immunosorbent assay (ELISA) was used to profile the EV biomarkers in the sera collected from 24 patients with CRC and 6 healthy donors (clinical information was shown in table S1). As shown in fig. S39A, no statistical difference was found in the average expression levels of CD9 between normal and CRC groups (*P* > 0.05), confirming that CD9 was unable to discriminate patients with CRC from healthy individuals. By contrast, the average expression level of GDF15 in patients with CRC was higher than that in healthy donors (*P* = 0.018) (fig. S39B), indicating that GDF15 has clinical potential as a biomarker for CRC diagnosis. The above results show that MB@CP-iPr holds high analytical features in enriching EVs from biofluids for downstream biomarker screening and identification.

## DISCUSSION

The high-efficiency isolation of EVs from various body fluids is key to their biomedical uses but remains a technical challenge. In this study, we report a reversible zwitterionic coordination strategy to achieve rapid, high-yield, and high-purity isolation of EVs from diverse biofluids. We found that CP, which is structurally inverse to PC but is otherwise virtually identical, can behave as an entirely powerful “hook” to capture EVs through specific CP-PC interactions. Moreover, the zwitterionic structure of CP provides favorable antifouling performance to the surface of MB@CPs, which effectively attenuates the contaminants of non-EV constituents throughout the whole isolation process. What excited us most is that the captured EVs could be efficiently released from the beads by only slightly increasing the solution temperature, without the addition of any reagents and complex operations. These features make MB@CP an invaluable tool for isolating EVs from clinical samples for downstream applications.

Purity, yield, recovery, and processing time are vital for evaluating an isolation technique. UC and density gradient centrifugation are two density-based techniques for EV isolation. However, the non-EV components with the similar density to EVs can be copelleted during centrifugation, resulting in low purity. In addition, centrifugation usually takes lengthy duration (8 to 20 hours) to achieve complete EV collection (table S2). Nevertheless, the yield and recovery are still limited. The PEG-based precipitation kits often show high isolation yield but poor purity because these kits could inevitably coprecipitate EVs with non-EV contaminants such as lipoproteins and RNA complexes. Immunomagnetic beads could capture EVs via antibody-antigen interactions. This immunology-based method is primarily limited by the heterogeneous expression of proteins on the EV membrane, resulting in the dropout of a considerable number of EVs. Moreover, the captured EVs were hardly released from the immunomagnetic beads. Our MB@CP-based isolation system ameliorates these intractable issues in EV isolation. The high-affinity PC-CP interactions allow MB@CPs to capture EVs with a high yield (>90%). The excellent antifouling performance and releasability of MB@CPs contribute respectively to the high purity (>90%) and recovery (>90%) of the captured EVs. Moreover, the isolation can be readily completed within 30 min, outperforming most reported approaches.

Impressively, the EVs released from MB@CPs maintain their intact biological structure and bioactivity, a prerequisite for subsequent analysis and engineering applications, including biomarker screening, EV drug delivery, and therapeutics. Comprehensive biomarker screening largely depends on omics-based technologies, such as proteomics and transcriptomics (*59*, *60*). As shown in this study, the EVs isolated by MB@CPs contain more useful biological signatures than UC in proteomics, demonstrating that MB@CPs are potentially helpful for biomarker discovery. With regard to EV engineering applications, the purity and bioactivity of the prepared EVs are crucial for achieving high therapy effects (*11*, *61*). The low purity of EVs may reduce the effectiveness of drug loading and result in possible toxicity (*62*). For instance, the biological activity of stem cell–derived EVs depends on the carried proteins and RNA components, and the contaminants that coexisted with EVs would impair EVs’ therapeutic effect (*63**)*. MB@CPs have performed well in isolating EVs from diverse unprocessed body fluids with high purity, holding eminent potential in assisting the scalable purification of clinical-grade EVs.

Despite the successful preparation of MB@CPs for rapid, high-yield, and high-purity isolation of EVs from diverse biofluids, this study also has limitations. First, although we isolated EVs from the culture media of four cell lines and investigated their protein expression with proteomics, only GDF15 was validated by dozens of clinical samples. Other newly identified biomarkers should be explored by large cohort studies. Second, the samples we used to isolate EVs (cell culture, serum, urine, and saliva) are still limited. More diverse body fluids, such as tear, cerebrospinal fluid, bile, and breast milk, are supposed to be adopted to illustrate the universality of MB@CPs in EV isolation and meet the demand of EV research in different settings.

Overall, we presented a powerful strategy to realize fast, high-yield, and high-purity isolation of EVs. Given that MB is a commercially available product, MB@CPs are prone to be productized for further industrial and clinical translation. Furthermore, this magnetic-based isolation is independent of cumbersome equipment and advantageous in fast speed and user-friendly processing. This reversible zwitterionic coordination strategy opens powerful vistas in EV isolation that will prompt both basic research and clinical applications of EVs.

## MATERIALS AND METHODS

### Cell culture

The SW480, SW620, DLD-1, and HIEC cells were purchased from the National Collection of Authenticated Cell Cultures (Shanghai, China). All cell lines were tested as negative for mycoplasma contamination. SW620 and HIEC cells were cultured in a Dulbecco's modified Eagle’s medium (DMEM) supplemented with 10% (v/v) FBS, penicillin (100 U ml^−1^), and streptomycin (100 μg ml^−1^) in a humidified atmosphere containing 5% CO_2_ at 37°C. The DLD-1 cells were cultured in a RPMI-1640 medium supplemented with 15% (v/v) FBS, penicillin (100 U ml^−1^), and streptomycin (100 μg ml^−1^) in a humidified atmosphere containing 5% CO_2_ at 37°C. The SW480 cells were cultured in an L15 medium (Gibco) supplemented with 15% (v/v) FBS, penicillin (100 U ml^−1^), and streptomycin (100 μg ml^−1^) in a humidified atmosphere of 0.1% CO_2_ at 37°C.

### Preparation of the model EVs

The SW620 cells were grown in nine 225-cm^3^ cell culture flasks (Corning) for 2 days until they reached 80% density. The cells were then cultured in FBS-free DMEM for 48 hours. The medium was collected and centrifuged at 300*g* for 5 min and 10,000*g* for 30 min and then treated through a 0.22-μm filter to remove dead cells and cellular detritus. Then, 450 ml of the medium was collected and continuously ultracentrifuged at 110,000*g* (Type 45 Ti angle rotor) and 4°C for 70 min. The pellet was collected by removing the supernatant, resuspended in PBS, and then centrifuged at 110,000*g* and 4°C for another 70 min. The EV pellets were suspended in PBS and stored at −80°C.

### Synthesis of five CP monomers

To avoid side products affecting the subsequent experimental results, all reactions were performed under absolute anhydrous conditions. All glassware was kept dry in an oven at 120°C and protected using dried argon at each step. Acetonitrile was dried by distillation against CaH_2_, and 2-(dimethylamino)ethyl methacrylate was dried with CaH_2_ and then distilled under reduced pressure. Tetrahydrofuran (THF) was dried by distillation with lithium aluminum hydride before use.

Next, 0.08 mol of extra dry methanol (or ethanol, isopropanol, methoxyethyl, and *n*-butyl alcohol), 0.12 mol of 2-(dimethylamino)ethyl methacrylate, and 200 mg of monomethyl ether hydroquinone (as an inhibitor) were added to a 100-ml Schlenk flask under dried argon atmosphere and cooled to −55°C. Subsequently, 0.05 mol of 2-chloro-2-oxo-1,3,2-dioxaphospholane was added dropwise over 2 hours. The reaction was continued for 8 hours, and the mixture was stirred at 25°C overnight. Next, the reaction mixture was cooled to −20°C, and the precipitate was filtered off using an air-free funnel and then directly filtered into a Schlenk flask under an argon atmosphere. The solution was stirred for 4 days at 70° to 75°C. An excess amount of dry methyl tert-butyl ether was added to the solution, and the precipitate was collected after vigorous stirring. The supernatant was removed by decantation, and the precipitate was further purified by repeated washing with THF until a clear supernatant was obtained. The typical yield of the different products was between 40 and 60%. The products were stored in methanol in the dark at −80°C.

### Preparation of MBs stabilized by sodium citrate

A modified solvothermal reaction was used to prepare MBs. Briefly, 2.2 g of FeCl_3_·6H_2_O, 3.6 g of NaAc, and 0.5 g of trisodium citrate dehydrate were sequentially dissolved in 60 ml of ethylene glycol with vigorous stirring. The homogeneous solution was transferred to a Teflon-lined stainless steel autoclave. The autoclave was heated to 220°C, maintained for 12 hours, and then cooled to room temperature to yield MBs, which were subsequently washed several times with ethanol and redispersed in ethanol for subsequent use. The as-synthesized MBs were measured to be ~400 nm in diameter.

### Modification of the MBs with double bond (C═C)

The MB surfaces were modified with γ-methacryloxypropyltrimethoxysilane (MPS) to form abundant double bonds (C═C). Briefly, a mixture of 45 ml of ethanol and 15 ml of isopropyl alcohol was added to 50 mg of the freshly-prepared MBs. Then, 3 ml of NH_3_·H_2_O and 0.45 ml of MPS were dropwise added to the solution under ultrasonic conditions. The mixture was stirred for 2 hours at 60°C. The products (MB-C═C) were washed several times with ethanol to remove excess materials.

### Synthesis of MB@CPs

The MB@CP core-shell structures were prepared via one-step distillation precipitation polymerization (DPP) of CP monomers in acetonitrile, with MBA as the cross-linker and AIBN as the initiator. MB-C═C (50 mg) was typically dispersed in 40 ml of acetonitrile in a clean 100-ml single-necked flask under ultrasonic conditions for 5 min. Then, a mixture of 200 mg of CP monomers, 20 mg of MBA, and 4.5 mg of AIBN was added to the flask to initiate polymerization. The flask was submerged in a heating oil bath attached to a fractionating column, a Liebig condenser, and a receiver. The reaction mixture was heated to the boiling state within 30 min by precisely controlling the heating temperature. The reaction was completed after 20 ml of acetonitrile was distilled from the reaction mixture in approximately 1 hour. The MB@CP microspheres were collected by magnetic separation and repeatedly washed with ethanol and water.

### Isolation of EVs using MB@CP-iPr

First, the model EVs derived from the SW620 cell line were fluorescently labeled with DiO (10 μM). Each model EV sample (100 μl) was then incubated with MB@CP-iPr (2 mg/ml) at room temperature for 30 min. The EVs were captured by the MBs, while the non-EV counterparts were removed by magnetic separation. The EV-bound MBs were rinsed three times with PBS, followed by characterizing their morphology with SEM.

### Thermal release of the captured EVs from MB@CP-iPr

The EV-bound MBs were tiled into a confocal dish and placed on a living cell workstation to adjust the temperature. The temperature was changed from 25° to 37°C or 42°C, and the release of the EVs was observed under a fluorescence confocal microscope after 10 min of temperature stabilization. The supernatants containing the released EVs were then collected for protein extraction. The release efficiency was calculated as the CD9 (EV marker) content extracted from the supernatant (released EVs) divided by the total content of CD9 from the captured EVs.

### Characterization of EVs

All characterizations of EVs follow the MISEV2018 guideline.

#### Measurement of particle sizes

The number and size distribution of the EVs were measured using NanoSight LM10 (Malvern). The EVs were diluted 1:1000 with PBS and then placed in the chamber to count the EV number and size distribution using NTA software.

#### Morphology characterization

The morphology of EVs was observed by a 200-kV cryo–transmission electron microscope (Talos F200C, Thermo Fisher Scientific, USA). Each EV sample was stained with 1% uranium dioxide acetate solution and dropped onto a copper grid.

### Wound healing assay

The solution (3 ml) of model EVs collected from the highly aggressive SW620 cells was divided into two parts, which were re-enriched with UC and MB@CP-iPr, respectively. The EV pellets of UC were suspended in 1.5 ml of L15 and then stored at 4°C. The EV-bound MB@CPs were arrested in 1.5 ml of the L15 medium, incubated at 42°C for 30 min to release the EVs, and then stored at 4°C. Note that the EVs should not be left at 4°C for more than 6 hours to prevent RNA or protein denaturation and inactivation. Approximately 5 × 10^5^ of SW480 cells in 15% FBS L15 were seeded into a confocal dish. The cells were incubated at 37°C in 0.1% CO_2_ until they reached ~90% confluence. Then, a pipette tip was used to scratch the cell monolayer. The medium was removed and washed three times with PBS to remove residual FBS and EVs from the medium. The DMEM medium containing the above-prepared EVs was added, and the wound width was monitored under a microscope at 0- and 24-hour time points.

### Cell uptake of EVs

The model EVs were prestained with DiO (10 μM); the excess DiO was washed away by ultrafiltration (100 kDa). The EV precipitate was resuspended using 3 ml of PBS and divided into two parts; then, the EVs were re-enriched with UC and MB@CP-iPr, respectively. Approximately 2 × 10^5^ of SW480 cells in 15% FBS L15 were seeded into a confocal dish and incubated overnight at 37°C in 0.1% CO_2_. After performing coculture for different durations at 37°C, the culture medium was discarded and replaced with L15 with DiI (10 μM) and Hoechst 33343 (10 μM) at 37°C for 20 min. Next, the medium was removed, and the cells were washed with PBS three times to remove excess DiI and Hoechst 33343 from the medium. The cell images were observed under a fluorescence confocal microscope equipped with a 60× oil immersion objective lens. The fluorescence excitation of DiI was fixed at 561 nm, and the emission was detected in the range of 570 to 620 nm. The fluorescence excitation of DiO was set at 486 nm, and the emission was observed in the field of 500 to 550 nm. The fluorescence excitation of Hoechst 33343 was set at 408 nm, and the emission was detected in the range of 425 to 475 nm.

### RNA extraction

Total EV RNA was extracted from the isolated EVs using the TRIzol Kit according to the manufacturer’s instructions. Briefly, 500 μl of TRIzol was added to the isolated EVs to lyse the EVs, and the total RNA was extracted by shaking vigorously with 200 μl of chloroform. After centrifugation at 12,000*g*, the upper water phase was transferred, and one-half volume of anhydrous ethanol was added. Last, the total RNA was enriched using an RNA adsorption column. A 15-μl aliquot of the RNA solution from each sample was placed on a 2% agarose gel. A standard RNA molecular weight range was used to estimate the RNA molecular weights, and the gels were stained with GelRed before analysis.

### Serum protein adsorption, purification, and SDS-PAGE experiments

A 0.5 ml of MB-C═C (2 mg/ml) and a series of MB@CPs were respectively concentrated by magnetic adsorption and then resuspended in 10% FBS (0.5 ml). The samples were incubated with sera for 2 hours at 25°C to allow protein adsorption. The samples were then washed with phosphate buffer containing 0.1% (v/v) Tween 20 (PBST) three times to remove the unbound proteins. The precipitates were resuspended in PBST and concentrated to ~50 μl. Next, 15 μl of 5× sample loading buffer was added to the pellets, followed by incubation at 70°C for 1 hour to release the adsorbed proteins. The resultant mixtures were centrifuged at 14,000*g* for 15 min to obtain supernatants containing released proteins. A 30-μl aliquot of the supernatant from each sample was reserved for SDS-PAGE. A standard protein molecular weight range was used to estimate protein molecular weights, and the gels were stained with Coomassie brilliant blue or silver before analysis.

### Yield and purity analysis of EVs isolated by UC or MB@CP-iPr

EV sample (50 μl; containing ~10^11^ EVs) was mixed with 950 μl of PBS, 10% FBS, or 100% FBS solutions to prepare the artificial biofluid samples. For UC isolation, different FBS solutions were diluted with PBS to 13 ml and then centrifuged at 110,000*g* (4°C) for 70 min using an ultracentrifuge (SW41 swing rotor) to yield the EV pellets. For MB@CP-iPr isolation, 0.5 ml of MB@CPs (2 mg/ml) was added into different FBS solutions and then incubated for 30 min at room temperature. After the isolation of EVs with UC or MB@CP-iPr, the EV pellets were resuspended in 100 μl of PBS for estimation of isolation yield and purity.

The isolation yield was used to analyze the loss of EVs during the different isolation procedures. For EVs yield analysis, an equal 30 μl of the resuspended EV pellets isolated by UC or MB@CP-iPr was simultaneously analyzed with Western blot by recording the expression of CD9, a typical EV biomarker. By comparing the band gray values between the isolated EVs (by UC or MB@CP-iPr) and the model EVs, the isolation yields of the two methods for the artificial biofluid samples were calculated as percentages.

The isolation purity was used to analyze the ratio between EVs and non-EV proteins in the EV pellets after being isolated by UC or MB@CP-iPr from biofluids. For EVs purity analysis, an equal 5 μg of protein of the resuspended EV pellets (measured by a BCA kit) isolated by UC or MB@CP-iPr was simultaneously analyzed with Western blot by recording the expression of CD9. By comparing the band gray values between the isolated EVs (by UC or MB@CP-iPr) and the model EVs, the isolation purities of the two methods for the artificial biofluid samples were calculated as percentages.

### Western blotting analysis

Western blotting was performed using 12% polyacrylamide gels in a Trans-Blot module, and the purified EV proteins were quantified using a BCA kit. The protein lysates were resolved by SDS-PAGE and transferred onto poly(vinylidene fluoride) membranes. The membranes were blocked with 5% skim milk for 30 min at room temperature and then incubated with primary antibodies overnight at 4°C. The following primary antibodies were used: anti-CD9, anti-CD63, anti-CD81, anti–APOB-1, anti–APOB-100, anti-UMOD, and anti-amylase. According to the product specifications, the dilution of the primary antibodies was 1:1000 to 1:5000. The membranes were washed five times for 5 min (1× tris-buffered saline with 0.5% Tween 20) and then incubated with horseradish peroxidase (HRP)–conjugated anti-mouse immunoglobulin G (IgG) or HRP-conjugated anti-rabbit IgG as the secondary antibody (1:2000) for 2 hours at 37°C. Then, the membranes were incubated with enhanced chemiluminescence for immunodetection. Last, chemiluminescent imaging was performed on a C600 system (Azure).

### MB@CP isolation of EVs from various biofluids

For the plasma sample, 300 μl of plasma was centrifuged at 3000*g* for 10 min at 4°C to remove cellular detritus and then 0.5 ml of MB@CPs (2 mg/ml) was added for EV isolation. Last, the EVs were released in 300 μl of PBS at 42°C for 30 min. Next, 10 ml of the urea sample was centrifuged at 5000*g* for 30 min at 4°C to remove cellular detritus, and then 1 ml of MB@CP (2 mg/ml) was added for EV isolation. Last, the EVs were released in 300 μl of PBS at 42°C for 30 min. In addition, 1 ml of the saliva sample was centrifuged at 3000*g* for 10 min at 4°C to remove cellular detritus, and then 0.5 ml of MB@CPs (2 mg/ml) was added for EV isolation. Last, the EVs were released in 300 μl of PBS at 42°C for 30 min. All biofluids were collected from healthy donors according to the manufacturer’s instructions using Schirmer’s test.

### UC isolation of EVs from various biofluids

The pretreatment procedures of the biofluid samples were the same as that described above. The samples were diluted with PBS to 13 ml and then centrifuged at 110,000*g* (4°C) for 70 min using an ultracentrifuge (SW41 swing rotor) to yield the EV pellets. The EV pellets were resuspended in 300 μl of PBS for further analysis.

### PEG isolation of EVs from various biofluids

The pretreatment procedures of the biofluid samples were also the same as that described above. The samples were mixed gently with the same volume of PEG solution and incubated overnight at 4°C. The solution was then centrifuged at 5000*g* for 30 min. The supernatant was discarded, and the EV pellets was resuspended in 300 μl of PBS for further analysis.

### Analysis of EV proteins by ELISA

The capture antibody (anti-CD63 antibody) was diluted with coating buffer to reach a final concentration of 0.9 μg/ml. Capture antibody (100 μl) was added to each microplate well and incubated at 4°C overnight. Then, the solution was discarded, followed by three times of PBST washing [0.2% Tween 20 in PBS (v/v)]. Blocking buffer (100 μl) [5% bovine serum albumin in PBS (w/v)] was added to each well and incubated at 37°C for 1 hour. After discarding the blocking buffer, 100 μl of EV sample was added and incubated at 4°C overnight, followed by another three-time washing with PBST. Subsequently, 100 μl of detection antibody (diluted with blocking buffer, 1.0 μg/ml for anti-CD63 antibody or anti-GDF15 antibody) was added and incubated at 37°C for 1 hour, followed by a three-time PBST washing operation. HRP-conjugated secondary antibody (100 μl) was added to each well (diluted with blocking solution, final concentration is 2 μg/ml) and incubated at 37°C for 40 min. The solution was discarded, and the plate was washed five times with PBST. Last, 200 μl of trimethylboron chemiluminescence chromogenic solution was added to each well; after reaction in the dark for 20 min, 50 μl of stop solution was added to each well. The absorbance at 450 nm was measured by a microplate reader.

## Supplementary Materials

### This PDF file includes:

General Information Figs. S1 to S39 Tables S1 and S2 References
