# Supplementary material for: Reversible zwitterionic coordination enables rapid, high-yield, and high-purity isolation of extracellular vesicles from biofluids
Source: Sci Adv. 2023 Apr 14;9(15):eadf4568. doi: 10.1126/sciadv.adf4568 (PMC10104463; doi:10.1126/sciadv.adf4568)
Supplement: Supplementary file 1 — General Information Figs. S1 to S39 Tables S1 and S2 References [file sciadv.adf4568_sm.pdf]

Science Advances
AAAS

Supplementary Materials for

Reversible zwitterionic coordination enables rapid, high-yield, and high-purity isolation of extracellular vesicles from biofluids

Qiang Li *et al.*

Corresponding author: Dingbin Liu, liudb@nankai.edu.cn

Sci. Adv. **9**, eadf4568 (2023)
DOI: 10.1126/sciadv.adf4568

This PDF file includes:

General Information
Figs. S1 to S39
Tables S1 and S2
References

General information

1.1 Chemicals and reagents

All chemical reagents were purchased from commercial suppliers and used without further purification. All of extra-dry material (include methanol, ethanol, isopropanol, methoxyethyl, *n*-butyl alcohol, 2-chloro-2-oxo-1,3,2-dioxaphospholane), 2-(dimethylamino) ethyl methacrylate, $FeCl_3 \cdot 6H_2O$, NaAc, trisodium citrate dehydrate, γ-methacryloxypropyltrimethoxysilane, bis-acrylamide, azodiisobutyronitrile, PEG was purchased from MERYER CO. LTD (Shanghai, China). DiI, DiO, Hoechst 33343, BCA Protein Quantification Kit, and BeyoECL Plus were obtained from Beyotime Biotechnology CO. Ltd. (Shanghai, China). Anti-Alix mouse monoclonal antibody (ab117600), anti-CD9 rabbit polyclonal antibody (ab223052), anti-CD81 rabbit monoclonal antibody (ab109201), antimouse IgG, HRP-linked antibody (ab205719) and antirabbit IgG, HRP-linked antibody (ab6721), anti-Salivary alpha amylase rabbit monoclonal antibody (ab201450), anti-UMOD rabbit monoclonal antibody (ab207170), anti-GDF15 rabbit monoclonal antibody (ab206414) were purchased from Abcam (Cambridge, MA). Anti-CD9 mouse monoclonal antibody (11029-MM01), anti-CD63 mouse monoclonal antibody (11271-MM03), anti CD81 mouse monoclonal antibody (14244-MM03), and anti-APOB1 rabbit polyclonal antibody (10686-T52) were purchased from Sino Biological (Beijing, China). Anti-APOB100 mouse monoclonal antibody (MAC003Hu22) was purchased from CLOUD-CLONE Coup. (Wuhan, China). Dulbecco's modified Eagle medium (DMEM), RPMI-1640 medium, L15 medium, fetal bovine serum (FBS), 0.25% trypsin-EDTA, and antibiotics (penicillin-streptomycin) were purchased from Gibco (Grand Island, NY). Dulbecco's phosphate-buffered saline (D-PBS) was purchased from Genview (TX, USA).

1.2 Instrumentation

^{1}H NMR spectra, and ^{35}P NMR spectra were carried out utilizing a Bruker AV 400 (400 MHz) instrument with CD_3OD as solvent. Mass spectra were carried out utilizing a VG ZAB-HS. FT-IR spectra were obtained by Nicolet iS50 (Thermofsher Scientific). Ultracentrifugation was performed using the Type 45Ti rotor or SW 41Ti rotor in an Optima XE-100 ultracentrifuge (Beckman Coulter). Fluorescence emission spectra were collected on a Hitachi F-4600 fluorimeter. The TEM images were obtained from an electron microscope (Talos F200C). The SEM images and energy dispersive spectra (EDS) were obtained from a FE-SEM (Apreo S LoVac). The TGA measurements were acquired from a TG8121 (Rigaku, Japan). The dynamic size and ζ-potential measurements were acquired from a Zetasizer (ZEN1690, Malvern). Particle concentration was analyzed by Nanoparticle tracking analyzer ZetaView PMX 110 (Particle Metrix, Meerbusch). Confocal fluorescence microscopy images were performed by Nikon A1+ confocal fluorescence microscope with a 100× lens. The gels of immunoblotting were observed with a gel image system (Azure c600, Azure Biosystems).

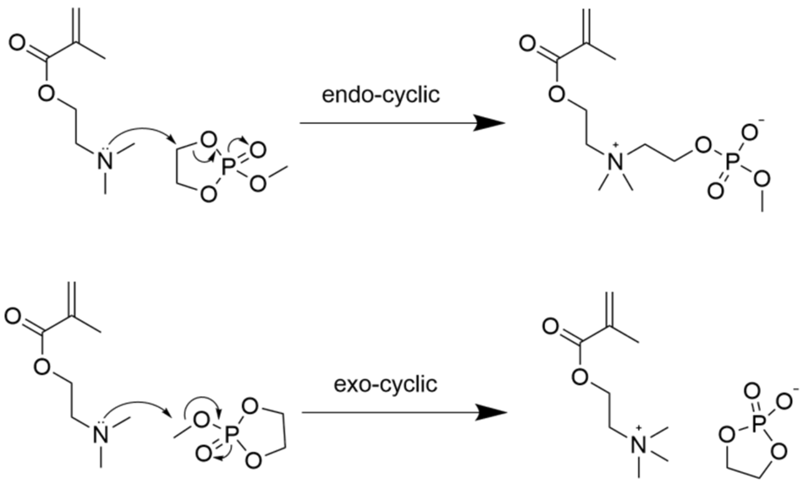

Figure S1. The ring open reaction of the Me-linked cyclic phosphate with tertiary amine is divided into exo-cyclic and endo-cyclic pathways.

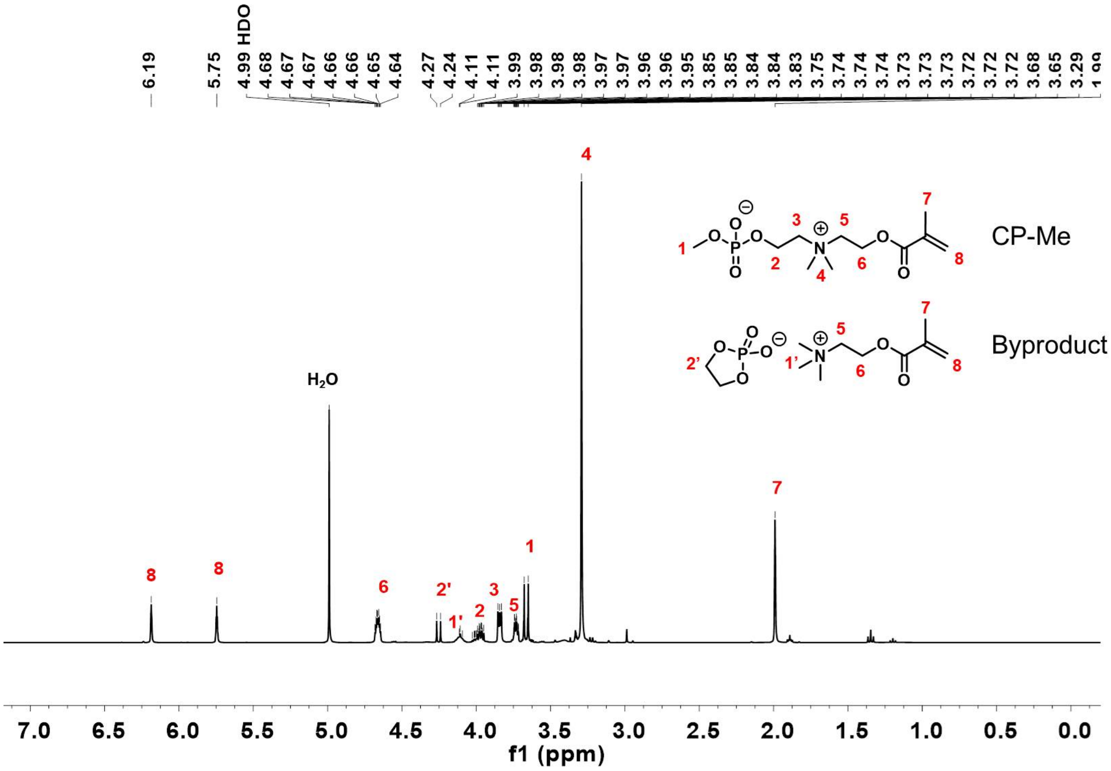

Figure S2. ^{1}H NMR spectrum of the mixture of CP-Me and its byproducts in CD_3OD. Since the target CP-Me and its byproduct are of identical molecular weight, chemical structure, and polarity, it is difficult to separate them using chromatography and selective precipitation. The peaks at 4.24 ppm (peak 2') and 4.11 ppm (peak 1') are respectively assigned to the methylene protons of the cyclic-phosphate anion and the methyl protons of the trimethyl-ammonium cation in the byproduct.

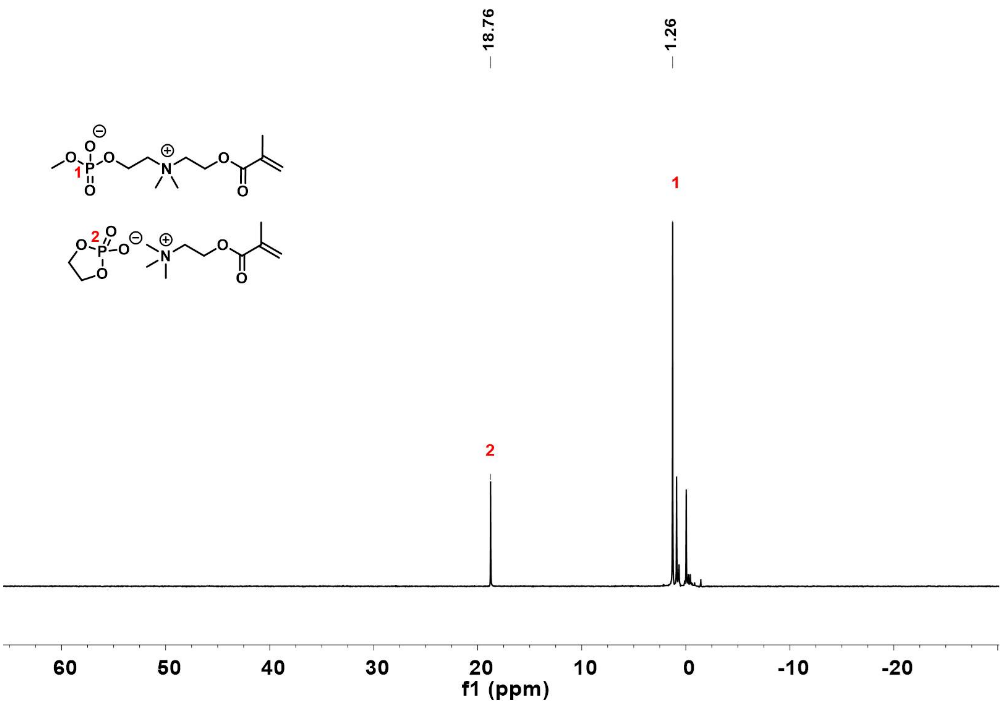

Figure S3. ^{31}P NMR spectrum of CP-Me and its byproducts in CD_3OD. ^{31}P NMR (δ: ppm, 162 MHz, Methanol-d_4): 18.76, 1.26. The signals at 18.76 ppm (peak 2) and 1.26 ppm (peak 1) are respectively assigned to the phosphorus in the cyclic-phosphate anion and CP-Me (reference 41 in the main text).

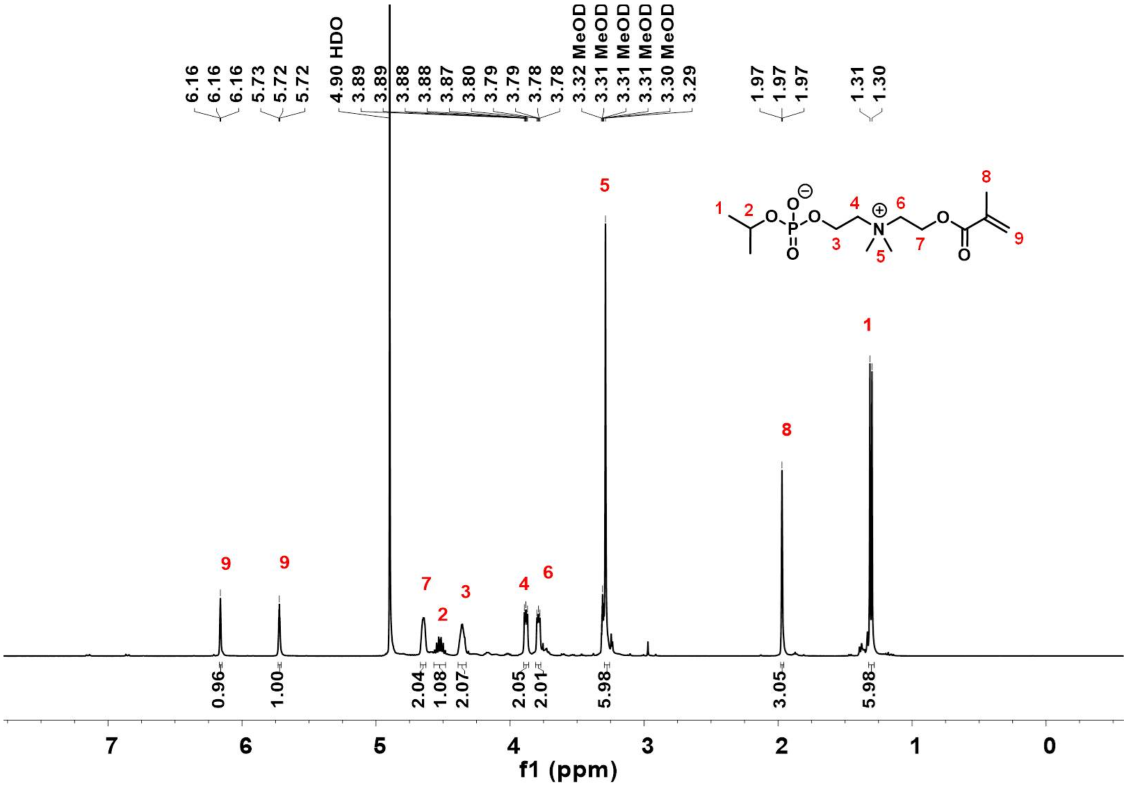

Figure S4. ^{1}H NMR spectrum of CP-iPr in CD_3OD. ^{1}H NMR (δ: ppm, 400 MHz, Methanol-d_4): 6.16 (s, 1H), 5.72 (s, 1H), 4.66 – 4.63 (m, 2H), 4.56 – 4.50 (m, 1H), 4.37 – 4.33 (m, 2H), 3.90 – 3.86 (m, 2H), 3.80 – 3.78 (m, 2H), 3.29 (s, 6H), 1.97 (t, J = 1.3 Hz, 3H), 1.31 (d, J = 6.2 Hz, 6H).

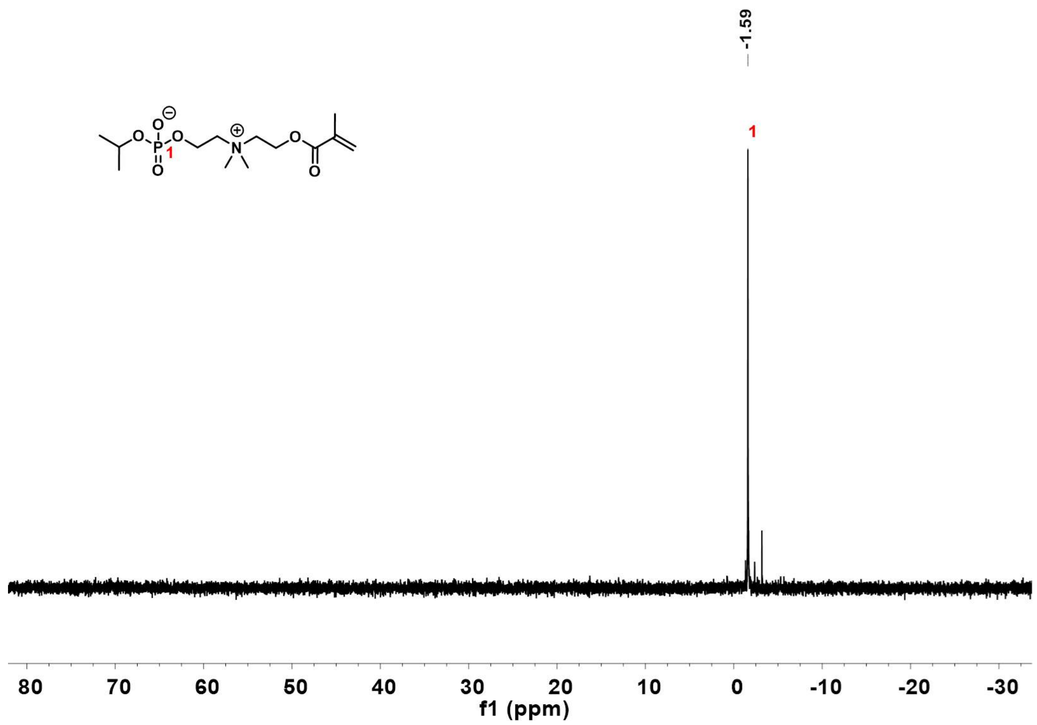

Figure S5. ^{31}P NMR spectrum of CP-iPr in CD_3OD. ^{31}P NMR (δ: ppm, 162 MHz, Methanol-d_4): -1.59.

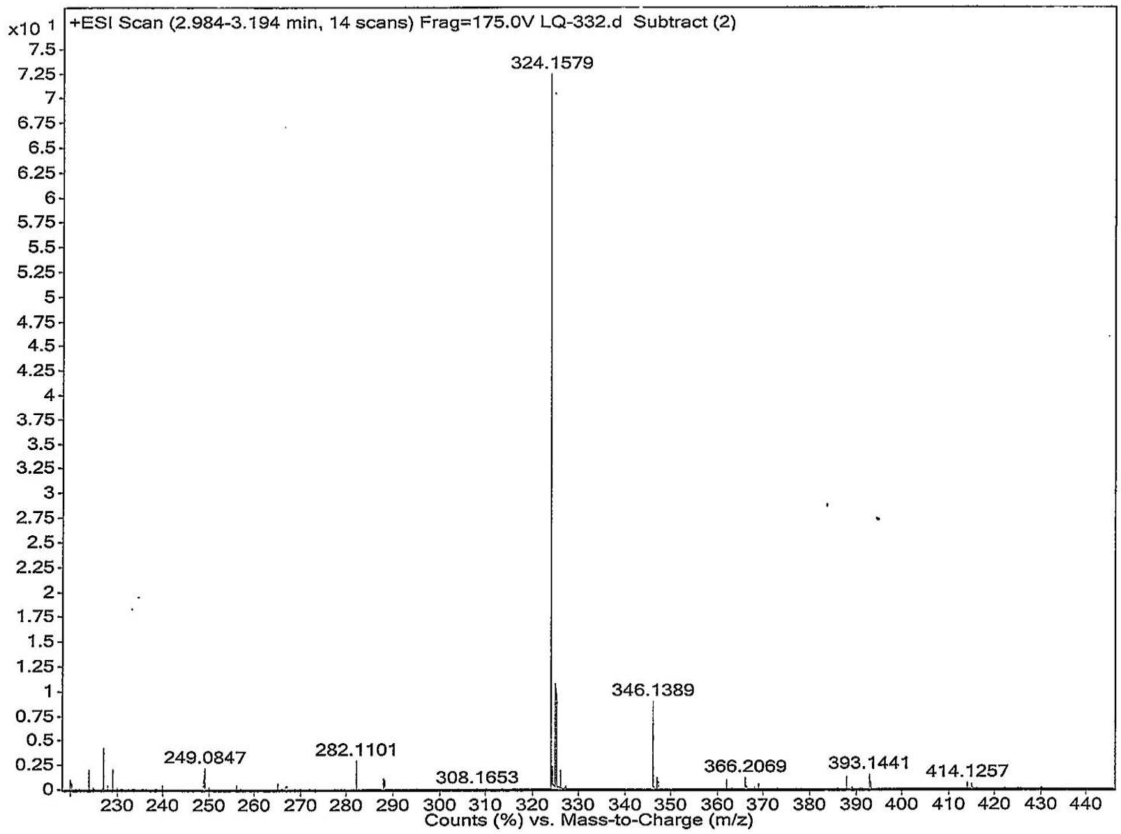

Figure S6. Mass spectrum of CP-iPr. ESI-MS: calc. for: $C_{13}H_{26}NO_6P$, $[M-H]^+$ 324.15, found 324.16.

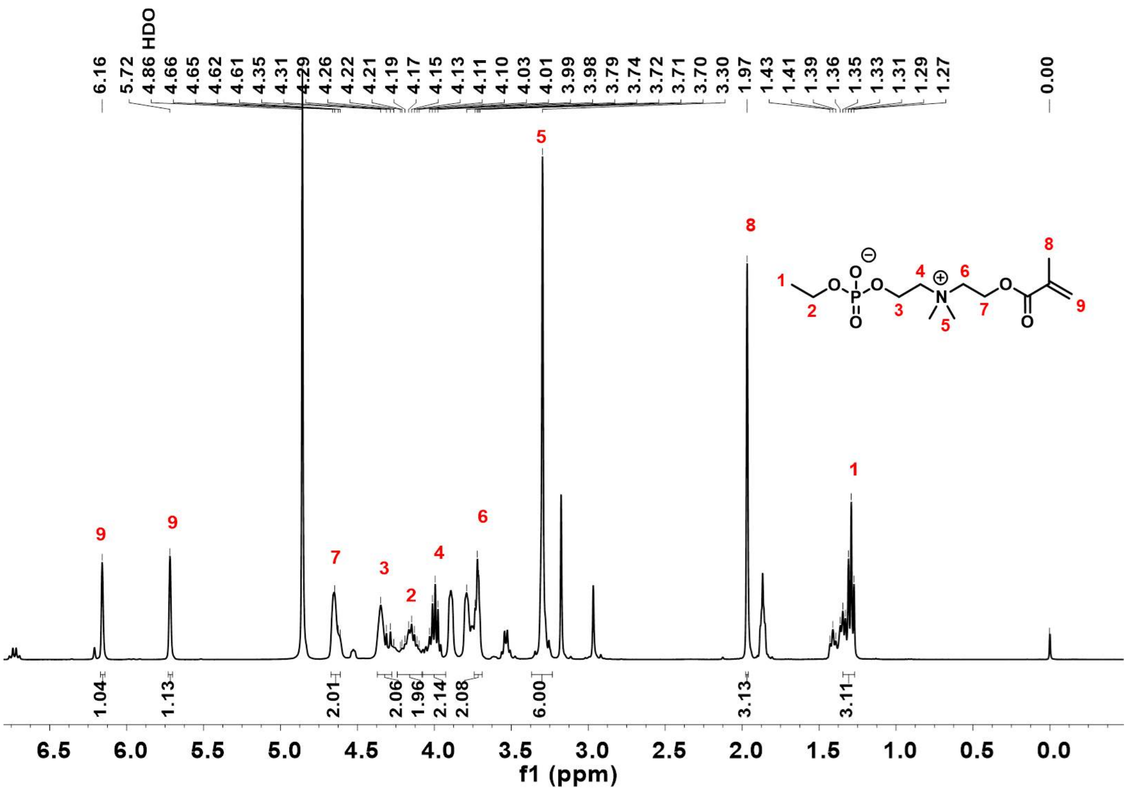

Figure S7. ^1H NMR spectrum of CP-Et in CD_3OD. ^1H NMR (δ: ppm, 400 MHz, Methanol-d_4): 6.16 (s, 1H), 5.72 (s, 1H), 4.66 – 4.61 (m, 2H), 4.35 (s, 2H), 4.22 – 4.13 (m, 2H), 3.99 – 3.91 (m, 2H), 3.72 (s, 2H), 3.30 (s, 6H), 1.97 (s, 3H), 1.36 – 1.27 (m, 3H).

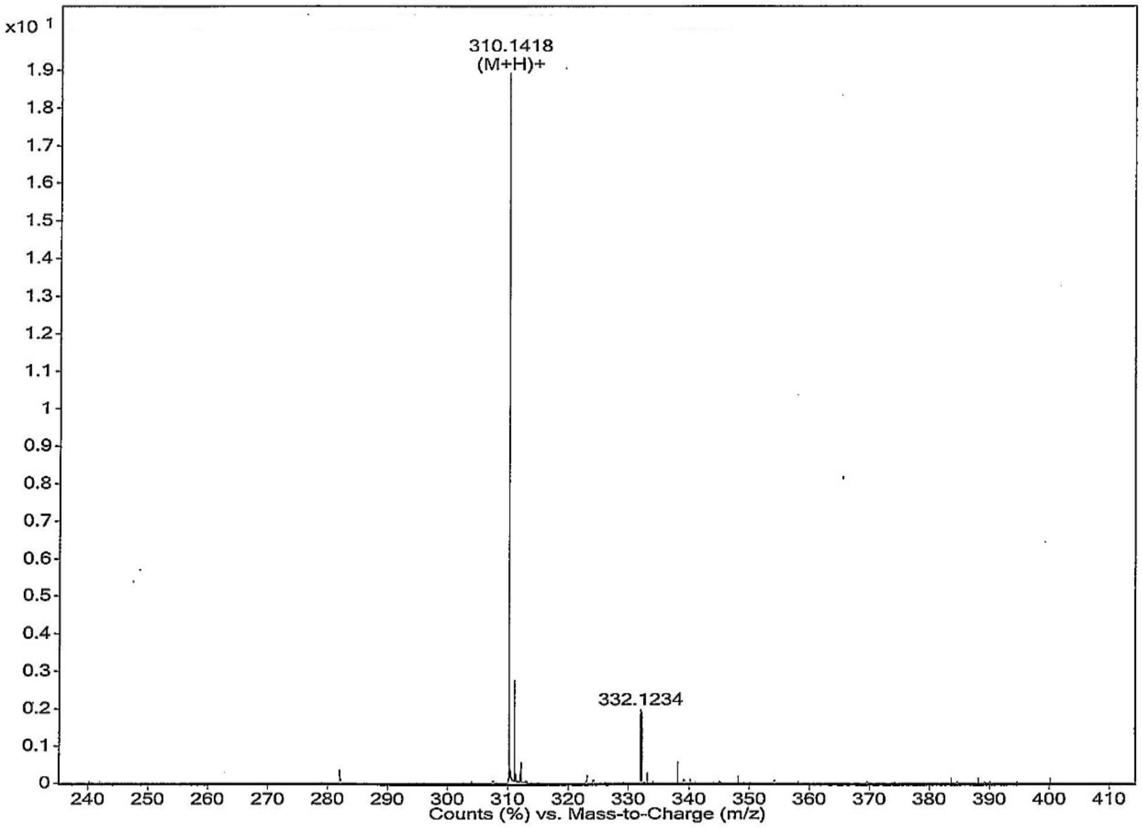

Figure S8. Mass spectrum of CP-Et. ESI-MS: calc. for: $C_{12}H_{24}NO_6P$, $[M-H]^+$ 310.13, found 310.14.

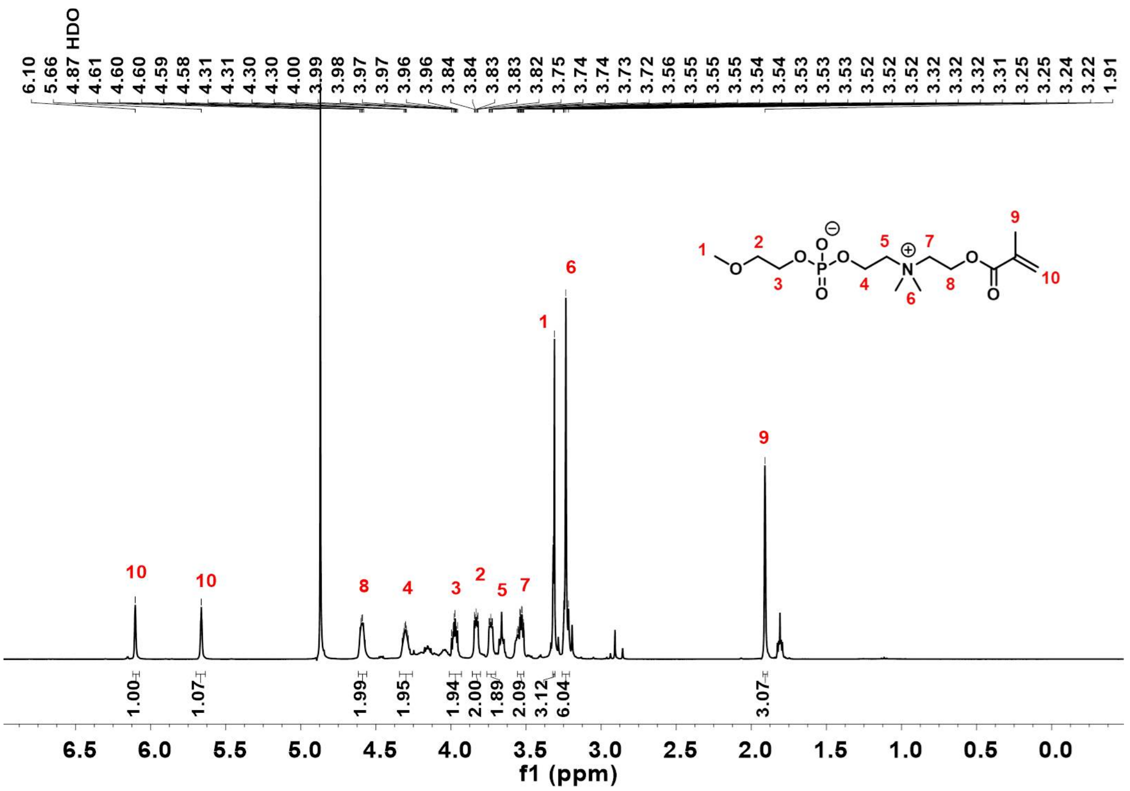

Figure S9. ^{1}H NMR spectrum of CP-MOE in CD_3OD. ^{1}H NMR (δ: ppm, 400 MHz, Methanol-d_4): 6.16 (s, 1H), 5.72 (s, 1H), 4.66 – 4.61 (m, 2H), 4.35 (s, 2H), 4.22 – 4.13 (m, 2H), 3.99 – 3.91 (m, 2H), 3.72 (s, 2H), 3.30 (s, 6H), 1.97 (s, 3H), 1.36 – 1.27 (m, 3H).

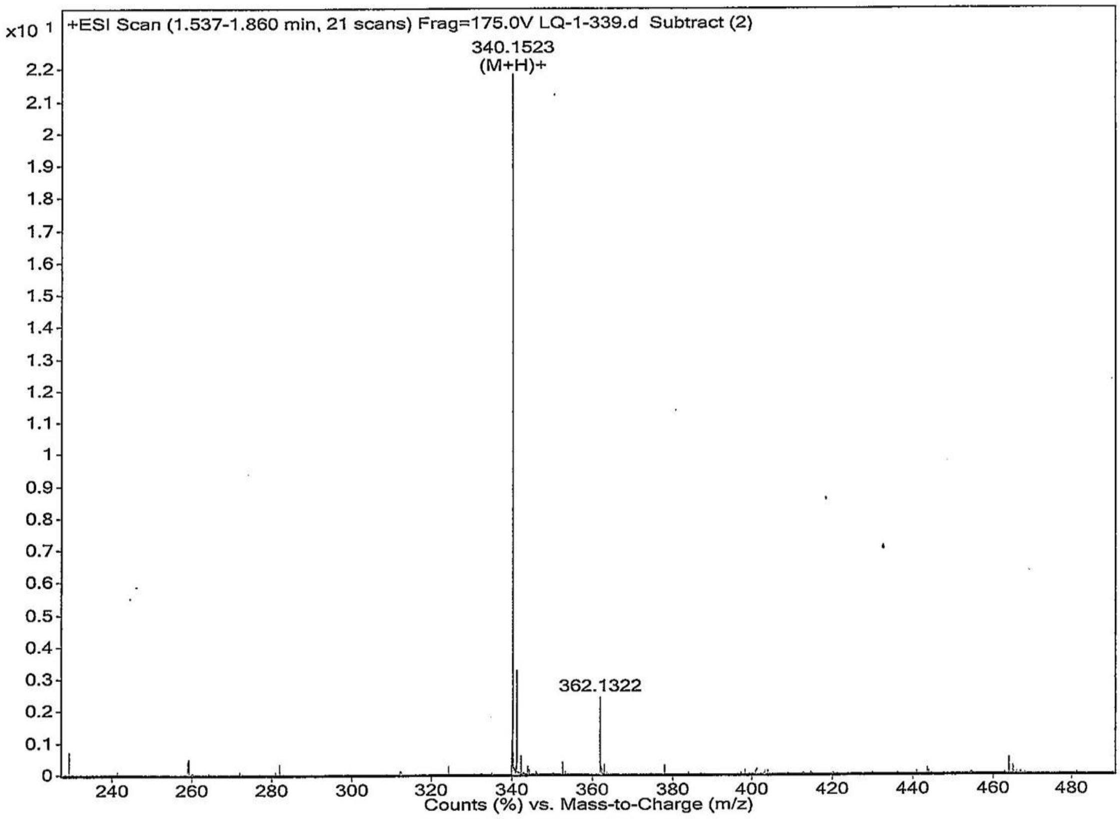

Figure S10. Mass spectrum of CP-MOE. ESI-MS: calc. for: $C_{13}H_{26}NO_7P$, $[M-H]^+$ 340.14, found 340.15.

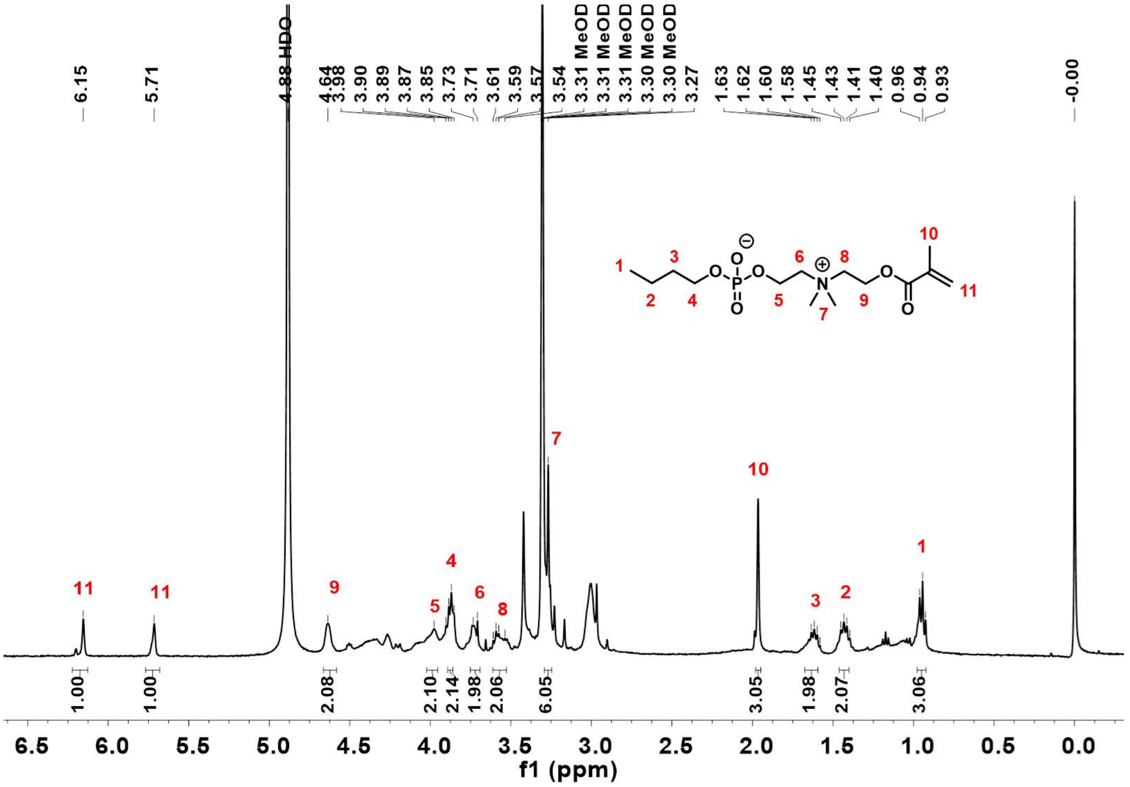

Figure S11. ^{1}H NMR spectrum of CP-*n*-butyl in CD_3OD. ^{1}H NMR (δ: ppm, 400 MHz, Methanol-d_4): 6.15 (s, 1H), 5.71 (s, 1H), 4.64 (s, 2H), 3.98 (s, 2H), 3.88 (d, J = 6.7 Hz, 2H), 3.72 (d, J = 10.5 Hz, 2H), 3.61 – 3.53 (m, 2H), 3.27 (s, 6H), 1.97 (s, 3H), 1.62 (t, J = 7.4 Hz, 2H), 1.46 – 1.40 (m, 2H), 0.94 (t, J = 7.3 Hz, 3H).

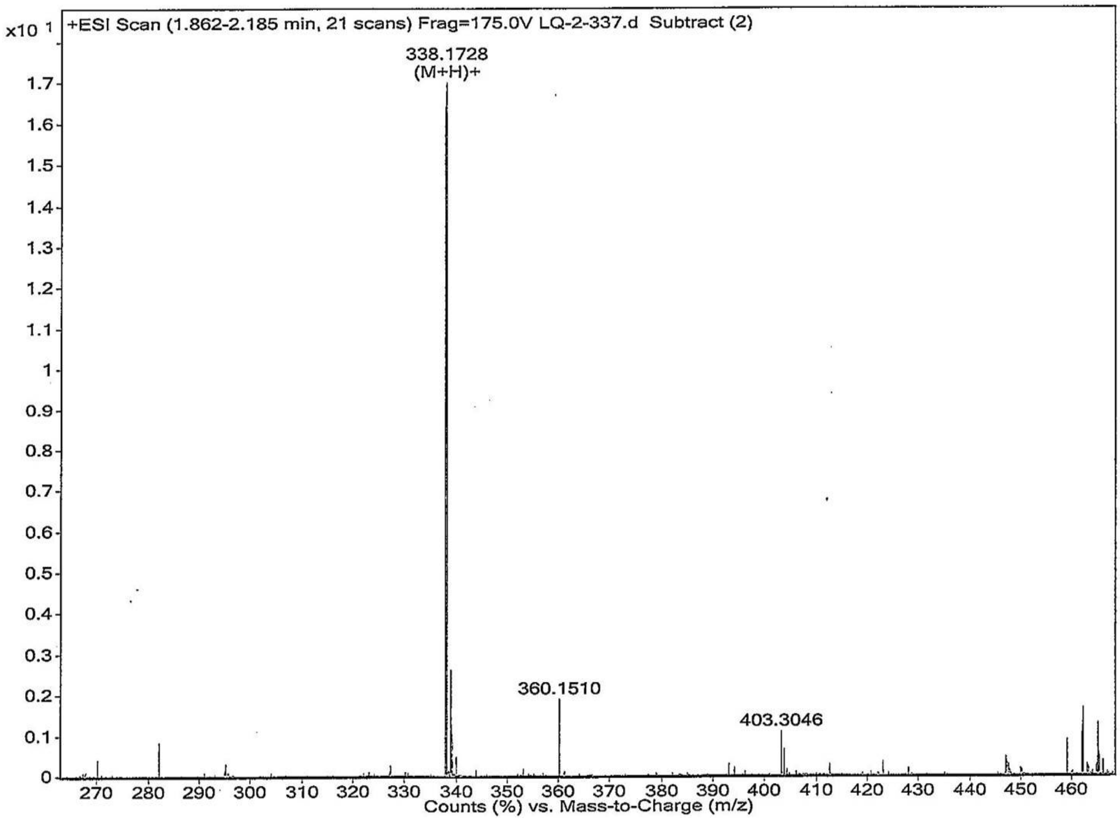

Figure S12. Mass spectrum of CP-*n*-butyl. ESI-MS: calc. for: $C_{14}H_{28}NO_6P$, $[M\text{-}H]^+$ 338.17, found 338.17.

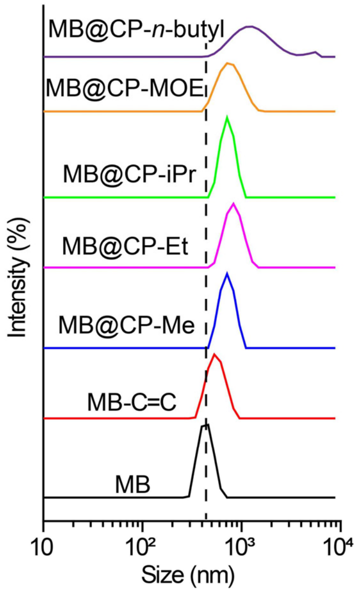

Fig S13. The DLS data of MB, MB-C=C, and MB@CPs bearing different headgroups (including Me, Et, iPr, MOE, and *n*-butyl). The size change of MBs can be used to evaluate the formation of MB@CPs and their mono-dispersity.

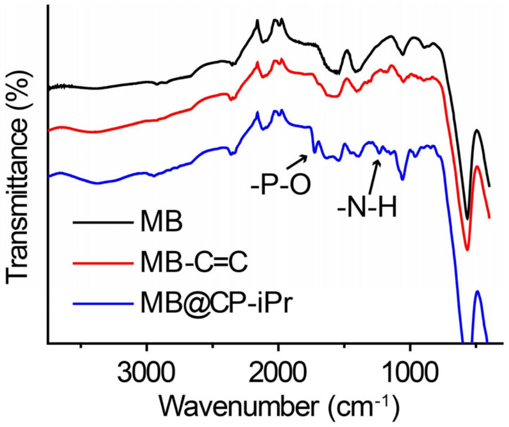

Figure S14. FT-IR spectra of MB, MB-C=C, and MB@CP-iPr. The peaks at 1725 cm^{-1} and 1234 cm^{-1} correspond to the P-O and N-H bonds in the nanogel shell of MB@CP-iPr.

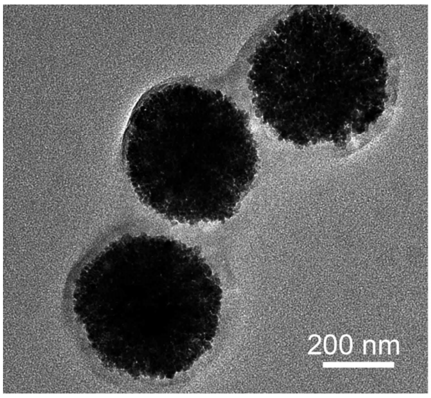

Figure S15. TEM image of MB@CP-iPr characteristic of a clear core-shell structure.

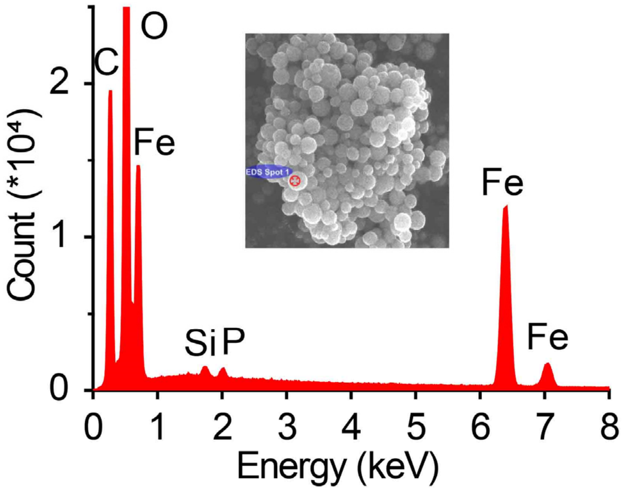

Figure S16. The EDS result shows the contents of Fe, Si, and P elements in the MB@CP-iPr, which correspond to the MB core, SiO_2 shell, and CP gel, respectively. Inner: a representative SEM image of MB@CP-iPr.

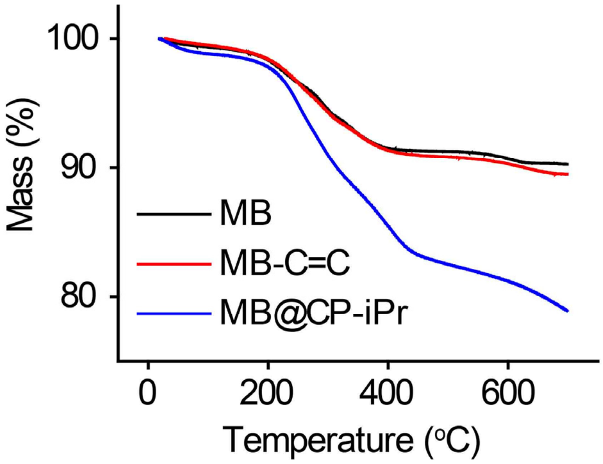

Figure S17. TGA of MB, MB-C=C, and MB@CP-iPr in the air atmosphere to evaluate the organic content of MB@CP-iPr.

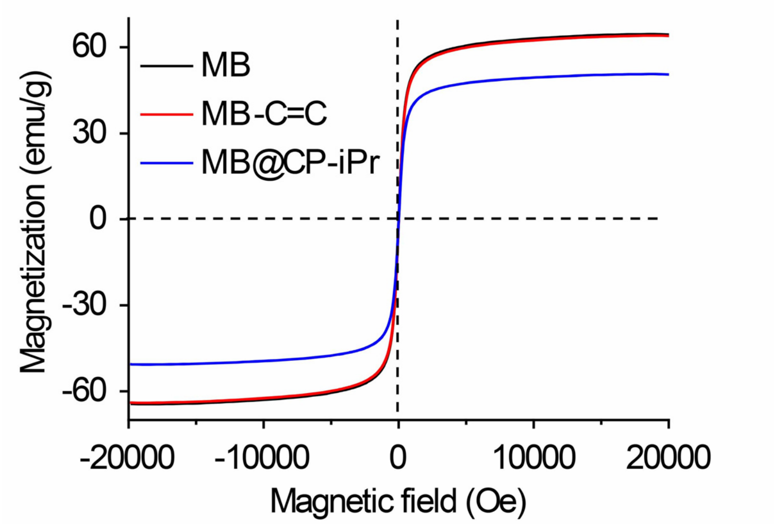

Figure S18. The magnetic hysteresis loop of MB, MB-C=C, and MB@CP-iPr. Oe, Oersted; emu, electromagnetic unit.

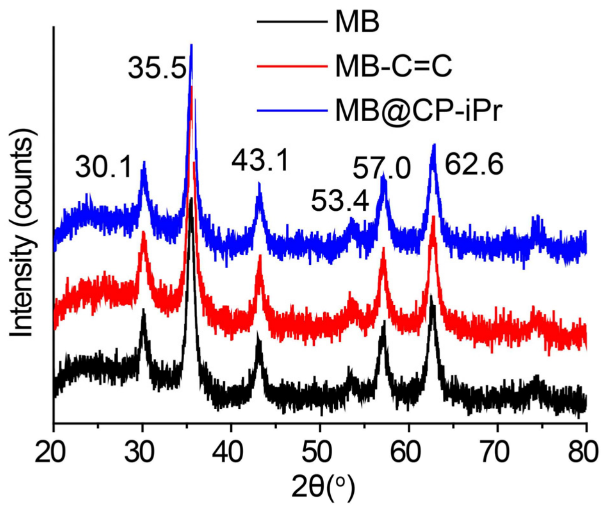

Figure S19. X-ray diffraction of MB, MB-C=C, and MB@CP-iPr. 2θ, diffraction angle.

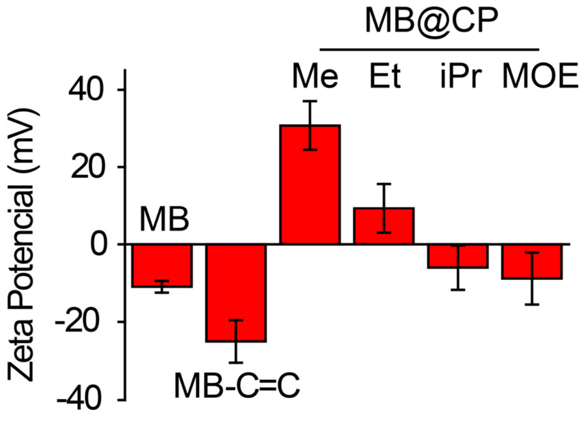

Figure S20. Zeta potentials of MB, MB-C=C, and MB@CPs bearing different headgroups. Error bars, mean±s.e.m (n=5).

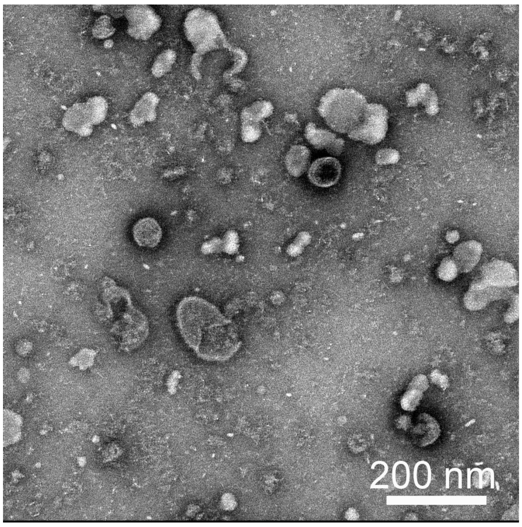

Figure S21. Cryo-EM image of the model SW620 EVs isolated by UC.

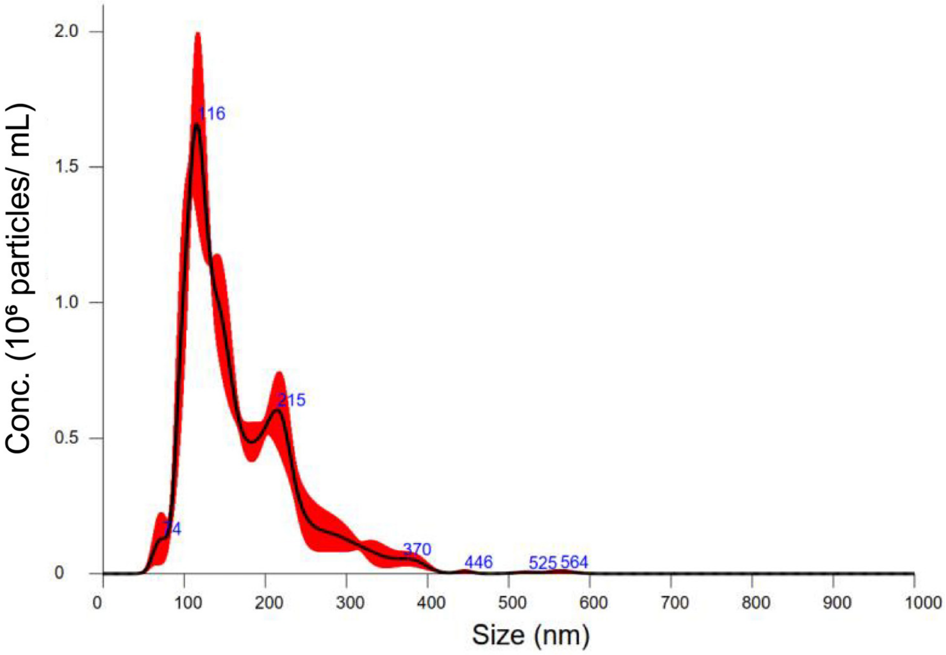

Figure S22. NTA measurement of the model SW620 EVs isolated by UC.

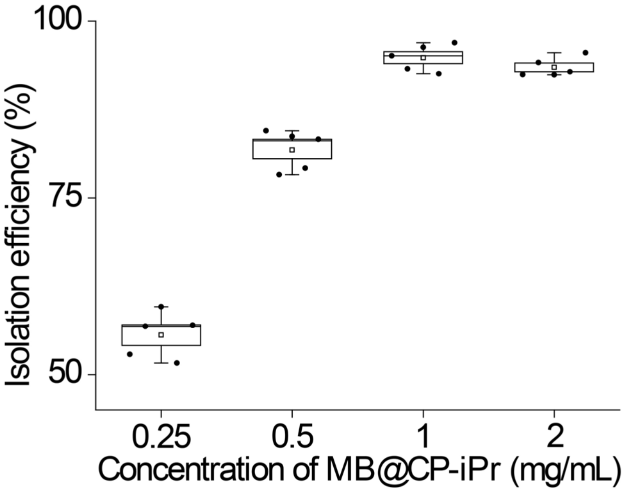

Figure S23. The plot of isolation efficiency versus different concentrations of MB@CP-iPr for isolating SW620 EVs from culture media. Error bars, mean±s.e.m (*n*=5).

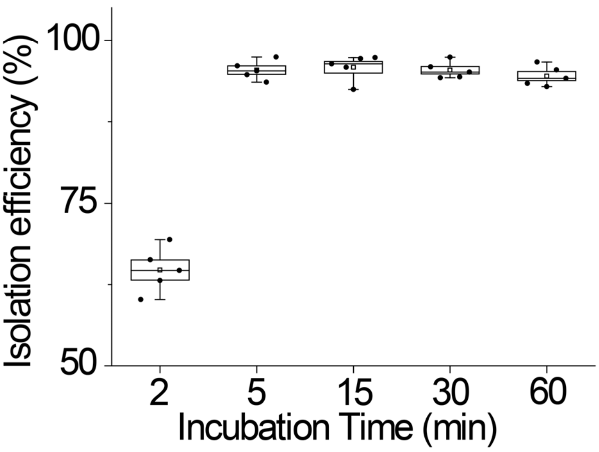

Figure S24. The plot of isolation efficiency versus different incubation periods for isolating SW620 EVs with MB@CP-iPr (1 mg/mL). Error bars, mean±s.e.m (*n*=5).

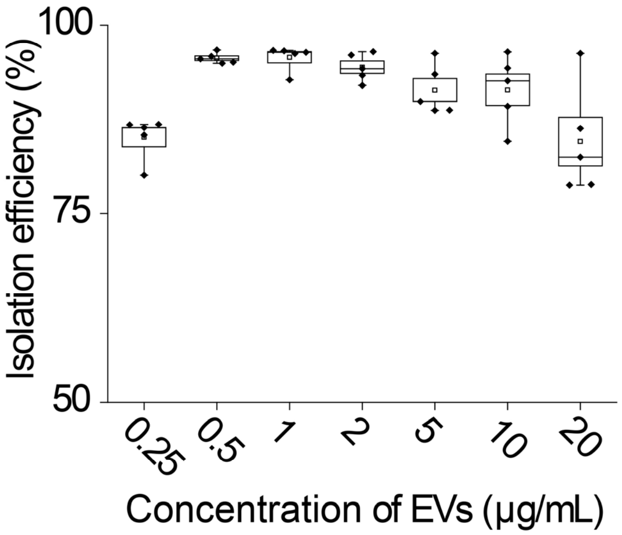

Figure S25. The plot of isolation efficiency versus different concentrations of EVs isolated by MB@CP-iPr (1 mg/mL). Error bars, mean±s.e.m (*n*=5).

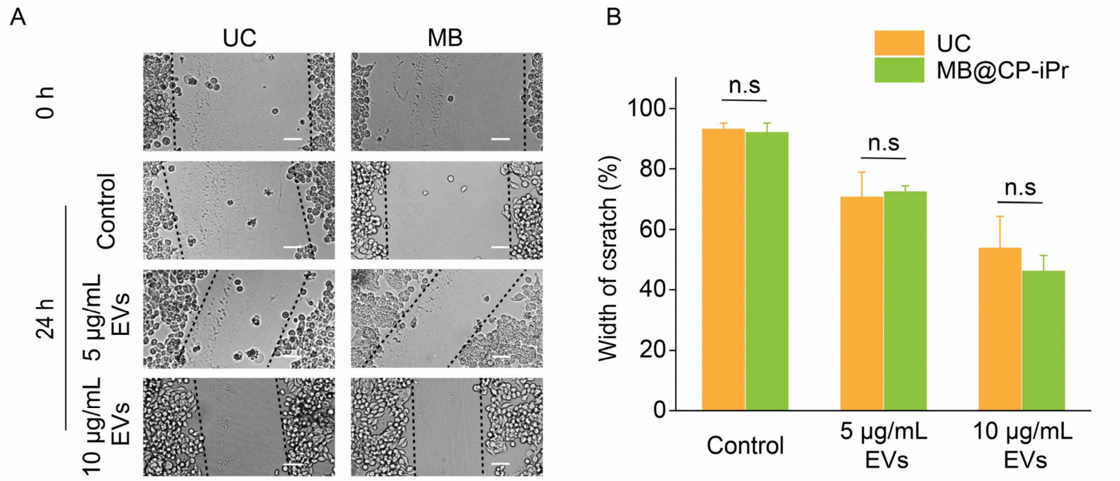

Figure S26. (A) Wound healing assays of SW480 cells incubated with SW620 EVs isolated by UC and MB at the same vesicle concentrations. Migration was assessed at 24 h time point after wounding. The absence of SW620 EVs was set as control. Scale bars: 100 μm. (B) The width of the scratches from the different groups in (A). Error bars, mean ± s.e.m (*n*=3, n.s means not significant or *P*>0.05).

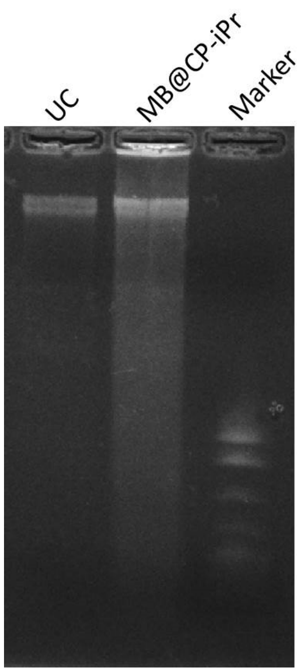

Figure S27. Agarose gel electrophoresis of total RNAs extracted from the EVs that were isolated by UC and MB@CP-iPr, respectively.

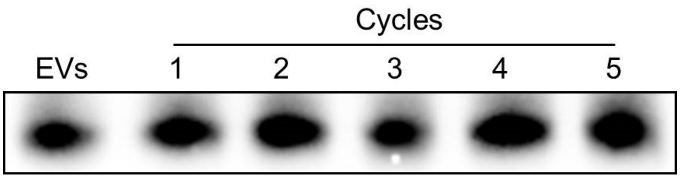

Figure S28. Western blotting analysis of CD9 expression on the model SW620 EVs isolated by MB@CP-iPr in five cycles.

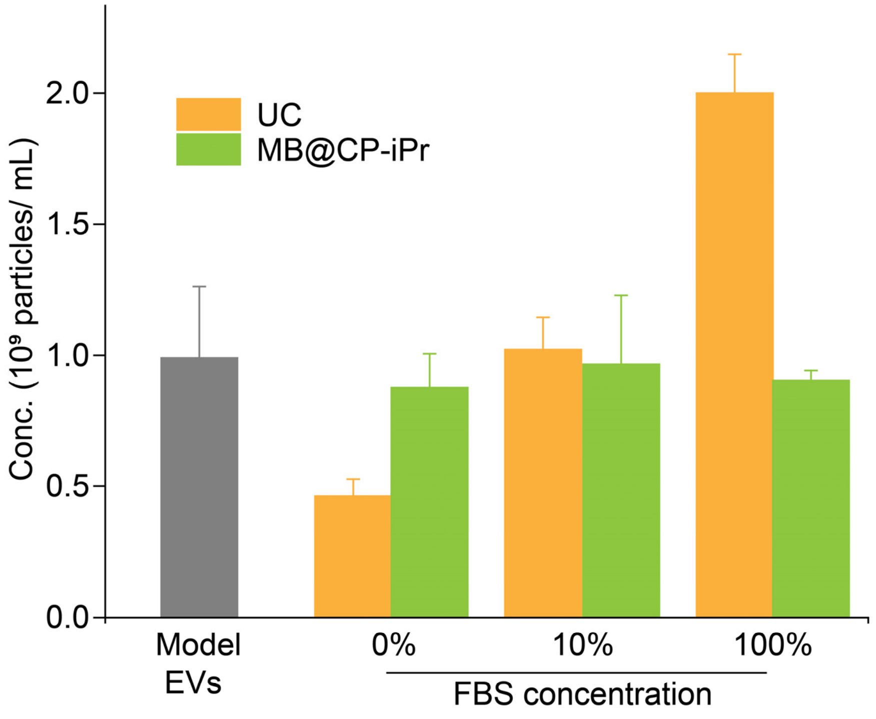

Figure S29. NTA analysis of the EV particle numbers in FBS at different concentrations (0%, 10%, and 100%) where the EVs were isolated by UC and MB@CP-iPr, respectively. Error bars, mean±s.e.m (*n*=3).

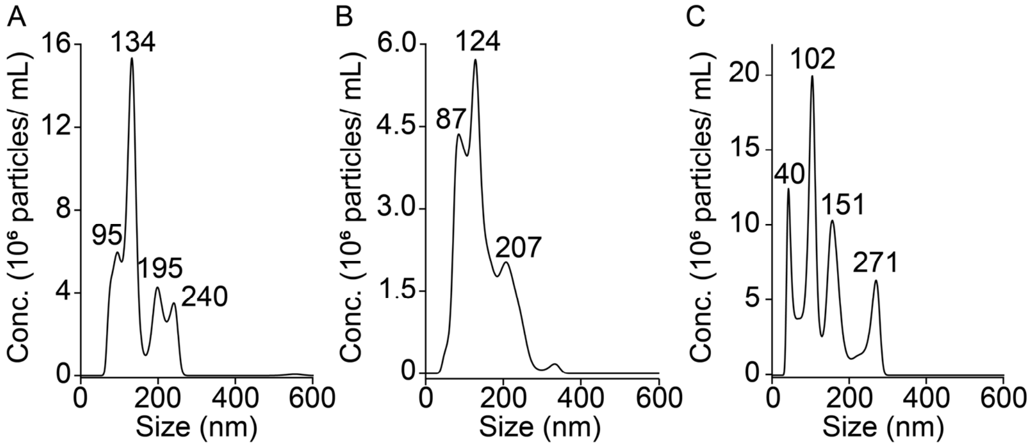

Figure S30. NTA measurement of the particle size distributions isolated by different methods. (A) The model EVs. The EVs isolated from 100% FBS by (B) MB@CP-iPr and (C) UC, respectively.

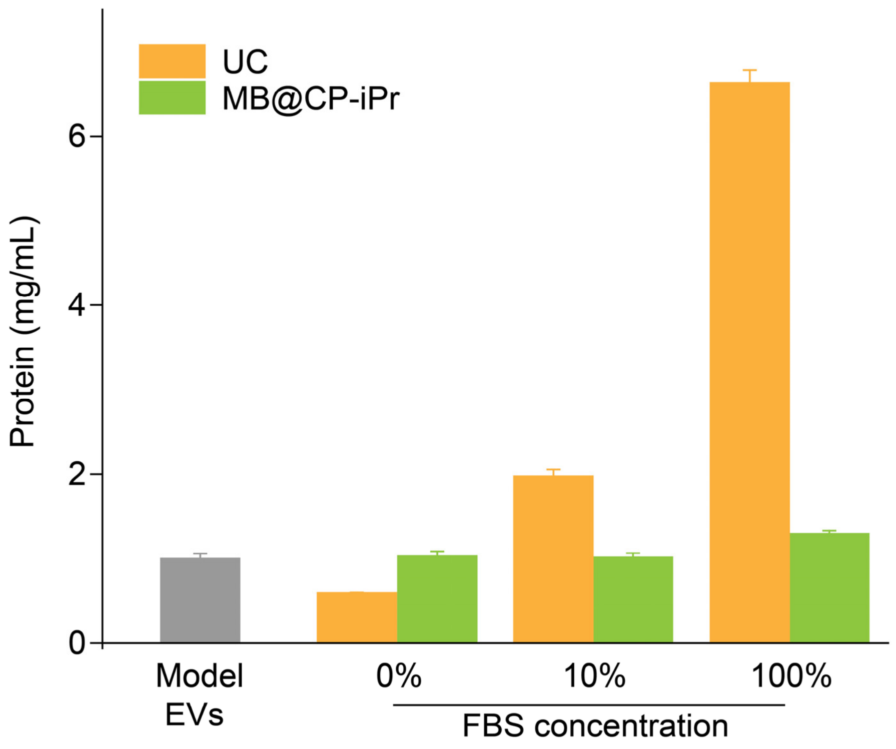

Figure S31. The protein concentrations of EV solutions in different FBS concentration groups (0%, 10%, and 100%) where the EVs were isolated by UC and MB@CP-iPr, respectively. Error bars, mean±s.e.m (n=3).

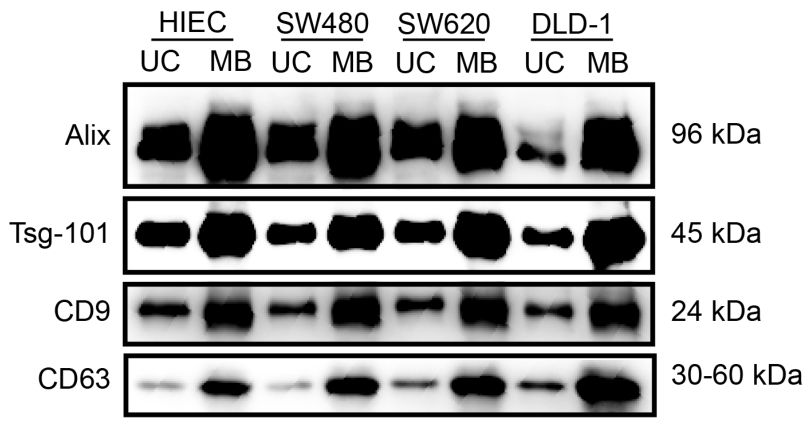

Figure S32. Western blotting analysis of the typical biomarkers of EVs derived from different cell lines. Each group was loaded with the same volume of EVs that were isolated by UC and MB@CP-iPr, respectively.

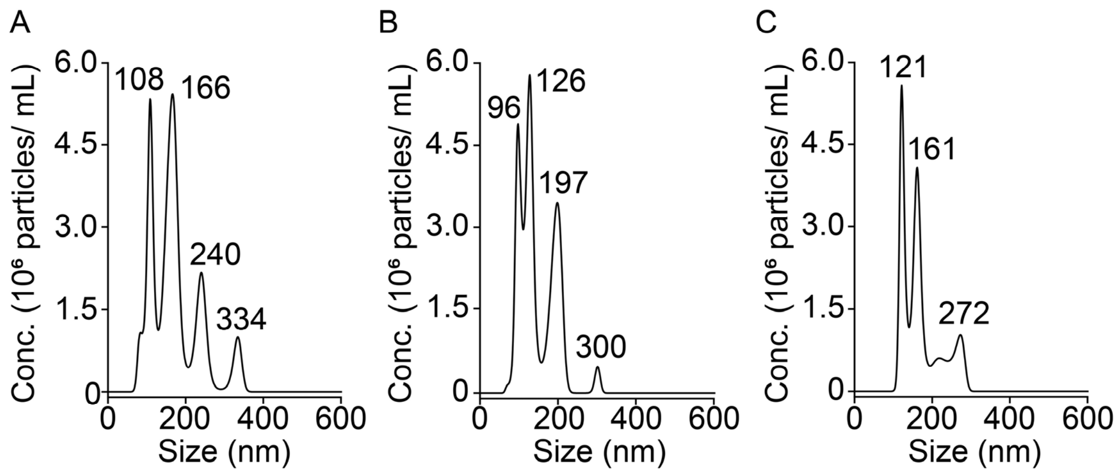

Figure S33. NTA measurement of the EVs derived from different cell lines that were isolated by MB@CP-iPr. (A) HIEC cell line. (B) SW480 cell line. (C) DLD-1 cell line.

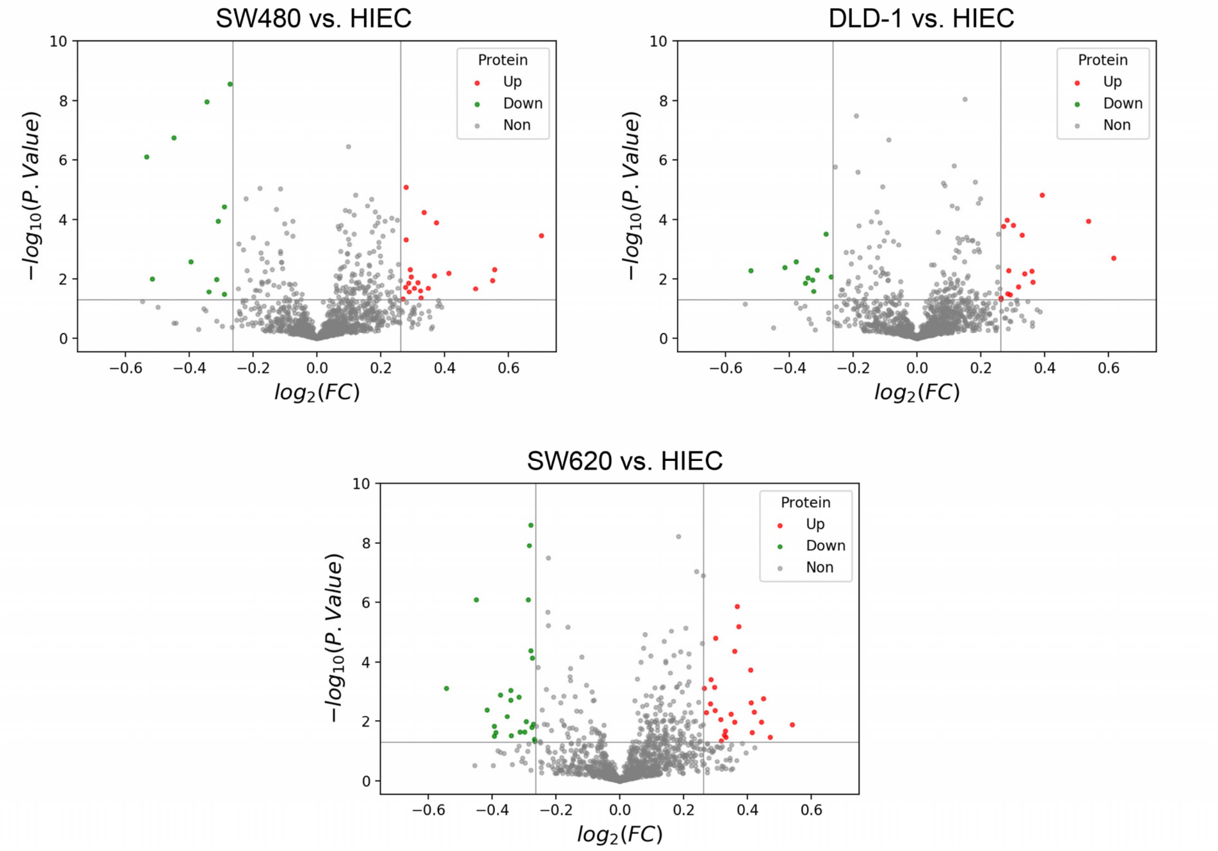

Figure S34. Volcano plot showing up-regulated proteins in 3 kinds of tumor cells. A false-discovery rate–corrected $P < 0.05$ and a fold change (FC) >1.5 were used to define upregulation (red) and downregulation (green).

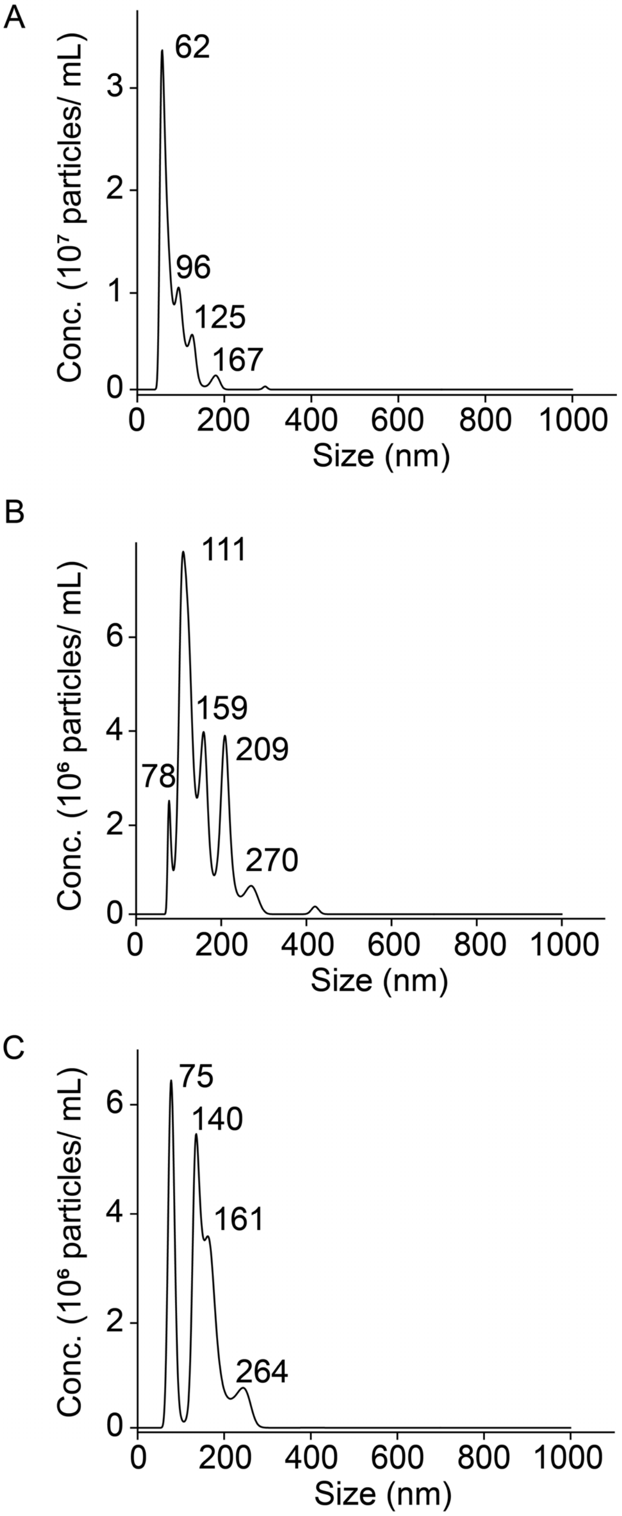

Figure S35. NTA measurement of the EVs isolated by MB@CP-iPr from different biofluids. (A) Serum. (B) Urine. (C) Saliva.

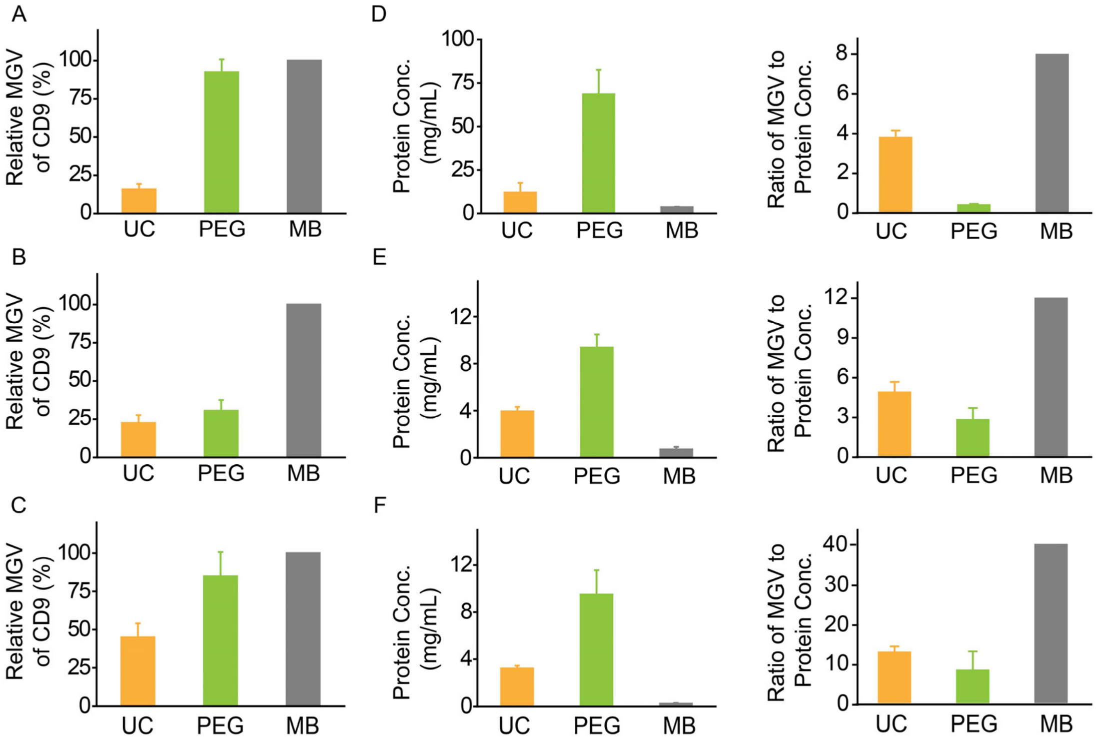

Figure S36. Relative mean gray values (MGVs) of the Western blotting bands of CD9 on the EVs after isolation from (**A**) human serum, (**B**) urine, and (**C**) saliva, respectively. The MGVs were obtained by ImageJ software. The concentrations of total enriched proteins of the EV samples after isolation from (**D**) human serum, (**E**) urine, and (**F**) saliva, respectively. The protein concentrations were determined by BCA kits. The MGVs relative to the corresponding total protein concentrations of the EV samples after isolation from (**G**) human serum, (**H**) urine, and (**I**) saliva, respectively, which are expressed as the ratio of MGV to protein concentration. These data are presented with three independent experiments. Error bars, mean±s.e.m (n=3).

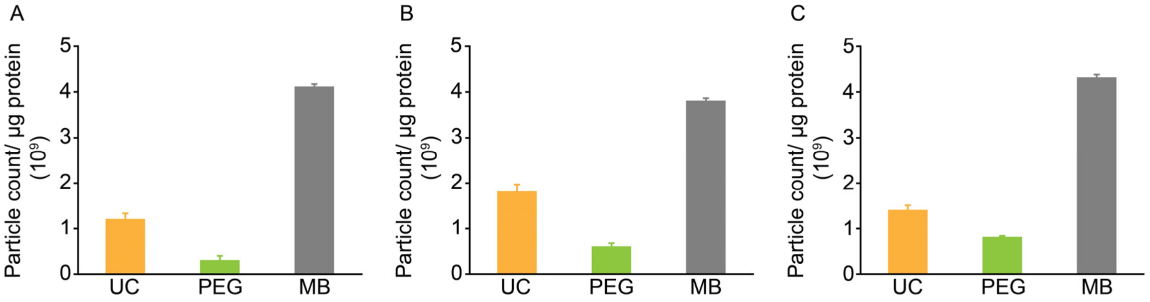

Figure S37. The ratios of EV particle count to the protein amount after isolating from (**A**) human serum, (**B**) urine, and (**C**) saliva, respectively. These data are presented with three independent experiments. Error bars, mean±s.e.m (*n*=3).

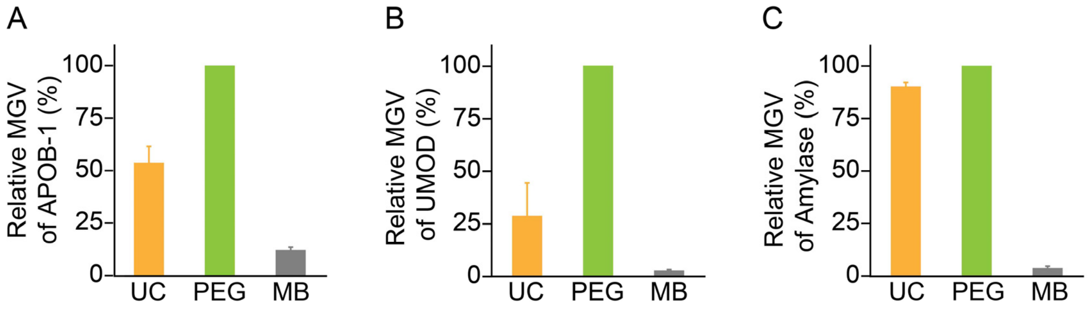

Figure S38. The relative levels of protein contaminations co-existed with EVs after isolating from (**A**) human serum, (**B**) urine, and (**C**) saliva, respectively. These data are presented with three independent experiments. Error bars, mean±s.e.m (*n*=3).

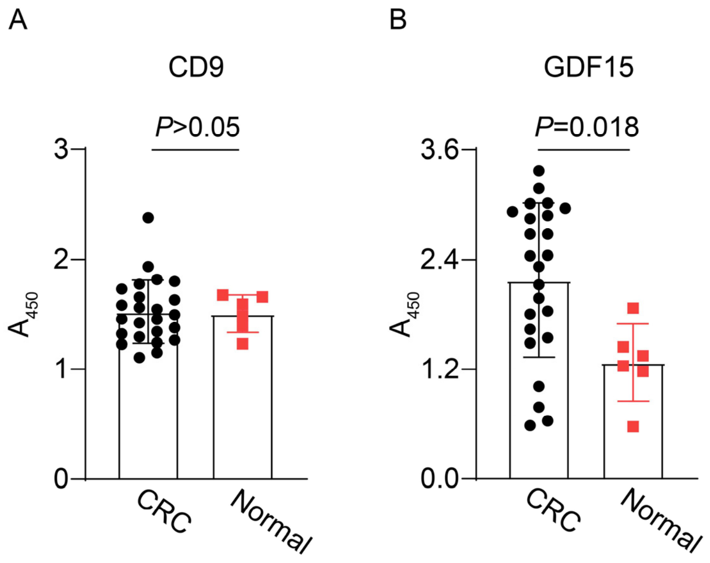

Figure S39. The scatter diagrams of CD9 (**A**) and GDF15 (**B**) levels on the EVs collected from CRC patients (*n*=24) and healthy donors (*n*=6).

Table S1. Clinical pathological parameters.

ID	Gender	Age	Dukes Stages*
1	Female	70	C
2	Male	66	B
3	Male	69	B
4	Male	62	C
5	Male	55	C
6	Male	52	B
7	Female	90	B
8	Female	36	C
9	Male	77	C
10	Male	64	C
11	Male	63	B
12	Male	81	C
13	Female	67	A
14	Female	63	C
15	Male	67	B
16	Male	63	C
17	Female	31	C
18	Female	66	C
19	Male	66	B
20	Male	64	C
21	Female	62	A
22	Male	61	B
23	Male	43	C
24	Male	72	B

*Dukes stages is a commonly-used clinical staging method for colorectal cancer. A means early stage; B means middle stage; C means late stage.

Table S2. Comparison of different EV isolation methods (references 64 and 65 in the main text**).**

Isolation Technique	Isolation Mechanism	Purity	Yield	Recovery	Sample Volume	Processing Time
Ultracentrifugation (UC)	Density	Low	Low	5-25%	100 s of mLs	8 h
Density-Gradient Centrifugation	Density	Low	Low	Intermediate	Up to 1 mL	20 h
Precipitation Kits	Surface Charge	Very low	High	Low	>100 µL	Overnight
Immunomagnetic Beads	Antibody-Antigen Interactions	High	> 50%	High	Up to 1 mL	2 h
MB@CP-iPr (This study)	CP-PC Interaction	> 90%	> 90%	> 90%	Up to 100 mL	< 30 min

REFERENCES AND NOTES

1. M. Colombo, G. Raposo, C. Théry, Biogenesis, secretion, and intercellular interactions of exosomes and other extracellular vesicles. *Annu. Rev. Cell Dev. Biol.* **30**, 255–289 (2014).

2. M. Harmati, M. Bukva, T. Böröczky, K. Buzás, E. Gyukity-Sebestyén, The role of the metabolite cargo of extracellular vesicles in tumor progression. *Cancer Metast. Rev.* **40**, 1203–1221 (2021).

3. G. Raposo, W. Stoorvogel, Extracellular vesicles: Exosomes, microvesicles, and friends. *J. Cell Biol.* **200**, 373–383 (2013).

4. H. Valadi, K. Ekström, A. Bossios, M. Sjöstrand, J. J. Lee, J. O. Lötvall, Exosome-mediated transfer of mRNAs and microRNAs is a novel mechanism of genetic exchange between cells. *Nat. Cell Biol.* **9**, 654–659 (2007).

5. Y. Liu, J. Fan, T. Xu, N. Ahmadinejad, K. Hess, S. H. Lin, J. J. Zhang, X. Liu, L. Liu, B. Ning, Z. X. Liao, T. Y. Hu, Extracellular vesicle tetraspanin-8 level predicts distant metastasis in non-small cell lung cancer after concurrent chemoradiation. *Sci. Adv.* **6**, eaaz6162 (2020).

6. T. Yasui, T. Yanagida, S. Ito, Y. Konakade, D. Takeshita, T. Naganawa, K. Nagashima, T. Shimada, N. Kaji, Y. Nakamura, I. A. Thiodorus, Y. He, S. Rahong, M. Kanai, H. Yukawa, T. Ochiya, T. Kawai, Y. Baba, Unveiling massive numbers of cancer-related urinary-microRNA candidates via nanowires. *Sci. Adv.* **3**, e1701133 (2017).

7. Y. Sun, Z. J. Xia, Z. Shang, K. B. Sun, X. M. Niu, L. Q. Qian, L. Y. Fan, C. X. Cao, H. Xiao, Facile preparation of salivary extracellular vesicles for cancer proteomics. *Sci. Rep.* **6**, 24669 (2016).

8. T. Takeuchi, K. Mori, H. Sunayama, E. Takano, Y. Kitayama, T. Shimizu, Y. Hirose, S. Inubushi, R. Sasaki, H. Tanino, Antibody-conjugated signaling nanocavities fabricated by dynamic molding for detecting cancers using small extracellular vesicle markers from tears. *J. Am. Chem. Soc.* **142**, 6617–6624 (2020).

9. M. Yanez-Mo, P. R. M. Siljander, Z. Andreu, A. B. Zavec, F. E. Borras, E. I. Buzas, K. Buzas, E. Casal, F. Cappello, J. Carvalho, E. Colas, A. Cordeiro-da Silva, S. Fais, J. M. Falcon-Perez, I. M. Ghobrial, B. Giebel, M. Gimona, M. Graner, I. Gursel, M. Gursel, N. H. H. Heegaard, A. Hendrix, P. Kierulf, K. Kokubun, M. Kosanovic, V. Kralj-Iglic, E.-M. Kramer-Albers, S. Laitinen, C. Lasser, T. Lener, E. Ligeti, A. Line, G. Lipps, A. Llorente, J. Lotvall, M. Mancek-Keber, A. Marcilla, M. Mittelbrunn, I. Nazarenko, E. N. M. Nolte-'t Hoen, T. A. Nyman, L. O'Driscoll, M. Olivan, C. Oliveira, E. Pallinger, H. A. Del Portillo, J. Reventos, M. Rigau, E. Rohde, M. Sammar, F. Sanchez-Madrid, N. Santarem, K. Schallmoser, M. S. Ostenfeld, W. Stoorvogel, R. Stukelj, S. G. Van der Grein, M. H. Vasconcelos, M. H. M. Wauben, O. De Wever, Biological properties of extracellular vesicles and their physiological functions. *J. Extracell. Vesicles* **4**, 27066 (2015).

10. J. D. Arroyo, J. R. Chevillet, E. M. Kroh, I. K. Ruf, C. C. Pritchard, D. F. Gibson, P. S. Mitchell, C. F. Bennett, E. L. Pogosova-Agadjanyan, D. L. Stirewalt, J. F. Tait, M. Tewari, Argonaute2 complexes carry a population of circulating microRNAs independent of vesicles in human plasma. *Proc. Natl. Acad. Sci. U.S.A.* **108**, 5003–5008 (2011).

11. S. El Andaloussi, I. Maeger, X. O. Breakefield, M. J. Wood, Extracellular vesicles: Biology and emerging therapeutic opportunities. *Nat. Rev. Drug Discov.* **12**, 347–357 (2013).

12. M. Tkach, C. Thery, Communication by extracellular vesicles: Where we are and where we need to go. *Cell* **164**, 1226–1232 (2016).

13. R. Kalluri, V. S. LeBleu, The biology, function, and biomedical applications of exosomes. *Science* **367**, eaau6977 (2020).

14. J. Matsuzaki, T. Ochiya, Circulating microRNAs and extracellular vesicles as potential cancer biomarkers: A systematic review. *Int. J. Clin. Oncol.* **22**, 413–420 (2017).

15. C. Liu, J. Zhao, F. Tian, J. Chang, W. Zhang, J. Sun, λ-DNA- and aptamer-mediated sorting and analysis of extracellular vesicles. *J. Am. Chem. Soc.* **141**, 3817–3821 (2019).

16. B. Lin, T. Tian, Y. Lu, D. Liu, M. Huang, L. Zhu, Z. Zhu, Y. Song, C. Yang, Tracing tumor-derived exosomal PD-L1 by dual-aptamer activated proximity-induced droplet digital PCR. *Angew. Chem. Int. Ed.* **60**, 7582–7586 (2021).

17. V. S. LeBleu, R. Kalluri, Exosomes as a multicomponent biomarker platform in cancer. *Trends Cancer* **6**, 767–774 (2020).

18. S. Amintas, V. Vendrely, C. Dupin, L. Buscail, C. Laurent, B. Bournet, J. P. Merlio, A. Bedel, F. Moreau-Gaudry, J. Boutin, S. Dabernat, E. Buscail, Next-generation cancer biomarkers: Extracellular vesicle DNA as a circulating surrogate of tumor DNA. *Front. Cell Dev. Biol.* **8**, 622408 (2021).

19. J. B. Simonsen, What are we looking at? Extracellular vesicles, lipoproteins, or both? *Circ. Res.* **121**, 920–922 (2017).

20. L. M. Millner, L. N. Strotman, The future of precision medicine in oncology. *Clin. Lab. Med.* **36**, 557–573 (2016).

21. O. P. B. Wiklander, M. A. Brennan, J. Lotval, X. O. Breakefield, S. El Andaloussi, Advances in therapeutic applications of extracellular vesicles. *Sci. Transl. Med.* **11**, eaav8521 (2019).

22. H. Choi, Y. Kim, A. Mirzaaghasi, J. Heo, Y. N. Kim, J. H. Shin, S. Kim, N. H. Kim, E. S. Cho, J. I. Yook, T. H. Yoo, E. Song, P. Kim, E. C. Shin, K. Chung, K. Choi, C. Choi, Exosome-based delivery of super-repressor IκBα relieves sepsis-associated organ damage and mortality. *Sci. Adv.* **6**, eaaz6980 (2020).

23. X. G. Zhang, E. G. F. Borg, A. M. Liaci, H. R. Vos, W. Stoorvogel, A novel three step protocol to isolate extracellular vesicles from plasma or cell culture medium with both high yield and purity. *J. Extracell. Vesicles* **9**, 1791450 (2020).

24. L. Dong, R. C. Zieren, K. Horie, C. J. Kim, E. Mallick, Y. Z. Jing, M. X. Feng, M. D. Kuczler, J. Green, S. R. Amend, K. W. Witwer, T. M. de Reijke, Y. K. Cho, K. J. Pienta, W. Xue, Comprehensive evaluation of methods for small extracellular vesicles separation

from human plasma, urine and cell culture medium. *J. Extracell. Vesicles* **10**, e12044 (2020).

25. Y. Wan, G. Cheng, X. Liu, S. J. Hao, M. Nisic, C. D. Zhu, Y. Q. Xia, W. Q. Li, Z. G. Wang, W. L. Zhang, S. J. Rice, A. Sebastian, I. Albert, C. P. Belani, S. Y. Zheng, Rapid magnetic isolation of extracellular vesicles via lipid-based nanoprobes. *Nat. Biomed. Eng.* **1**, 0058 (2017).

26. X. F. Wu, L. Li, A. Iliuk, W. A. Tao, Highly efficient phosphoproteome capture and analysis from urinary extracellular vesicles. *J. Proteome Res.* **17**, 3308–3316 (2018).

27. C. Liu, J. X. Zhao, F. Tian, L. L. Cai, W. Zhang, Q. Feng, J. Q. Chang, F. N. Wan, Y. J. Yang, B. Dai, Y. L. Cong, B. Q. Ding, J. S. Sun, W. H. Tan, Low-cost thermophoretic profiling of extracellular-vesicle surface proteins for the early detection and classification of cancers. *Nat. Biomed. Eng.* **3**, 183–193 (2019).

28. Y. C. Chen, Q. F. Zhu, L. M. Cheng, Y. Wang, M. Li, Q. S. Yang, L. Hu, D. D. Lou, J. Y. Li, X. J. Dong, L. P. Lee, F. Liu, Exosome detection via the ultrafast-isolation system: EXODUS. *Nat. Methods* **18**, 212–218 (2021).

29. E. Oeyen, K. Van Mol, G. Baggerman, H. Willems, K. Boonen, C. Rolfo, P. Pauwels, A. Jacobs, K. Schildermans, W. C. Cho, I. Mertens, Ultrafiltration and size exclusion chromatography combined with asymmetrical-flow field-flow fractionation for the isolation and characterisation of extracellular vesicles from urine. *J. Extracell. Vesicles* **7**, 1490143 (2018).

30. J. Z. Nordin, Y. Lee, P. Vader, I. Mager, H. J. Johansson, W. Heusermann, O. P. B. Wiklander, M. Hallbrink, Y. Seow, J. J. Bultema, J. Gilthorpe, T. Davies, P. J. Fairchild, S. Gabrielsson, N. C. Meisner-Kober, J. Lehtio, C. I. E. Smith, M. J. A. Wood, S. E. L. Andaloussi, Ultrafiltration with size-exclusion liquid chromatography for high yield isolation of extracellular vesicles preserving intact biophysical and functional properties. *Nanomedicine* **11**, 879–883 (2015).

31. C. Tripisciano, R. Weiss, T. Eichhorn, A. Spittler, T. Heuser, M. B. Fischer, V. Weber, Different potential of extracellular vesicles to support thrombin generation: Contributions of phosphatidylserine, tissue factor, and cellular origin. *Sci. Rep.* **7**, 6522 (2017).

32. R. C. Lai, F. Arslan, M. M. Lee, N. S. K. Sze, A. Choo, T. S. Chen, M. Salto-Tellez, L. Timmers, C. N. Lee, R. M. El Oakley, G. Pasterkamp, D. P. V. de Kleijn, S. K. Lim, Exosome secreted by MSC reduces myocardial ischemia/reperfusion injury. *Stem Cell Res.* **4**, 214–222 (2010).

33. M. Grey, C. J. Dunning, R. Gaspar, C. Grey, P. Brundin, E. Sparr, S. Linse, Acceleration of α-Synuclein aggregation by exosomes. *J. Biol. Chem.* **290**, 2969–2982 (2015).

34. X. F. Yu, Z. H. Liu, J. Janzen, I. Chafeeva, S. Horte, W. Chen, R. K. Kainthan, J. N. Kizhakkedathu, D. E. Brooks, Polyvalent choline phosphate as a universal biomembrane adhesive. *Nat. Mater.* **11**, 468–476 (2012).

35. X. F. Yu, X. Q. Yang, S. Horte, J. N. Kizhakkedathu, D. E. Brooks, ATRP synthesis of poly(2-(methacryloyloxy)ethyl choline phosphate): A multivalent universal biomembrane adhesive. *Chem. Commun.* **49**, 6831–6833 (2013).

36. X. Y. Chen, T. C. Chen, Z. F. Lin, X. E. Li, W. Wu, J. S. Li, Choline phosphate functionalized surface: Protein-resistant but cell-adhesive zwitterionic surface potential for tissue engineering. *Chem. Commun.* **51**, 487–490 (2015).

37. J. B. Schlenoff, Zwitteration: Coating surfaces with zwitterionic functionality to reduce nonspecific adsorption. *Langmuir* **30**, 9625–9636 (2014).

38. R. A. Letteri, C. F. Santa Chalarca, Y. Bai, R. C. Hayward, T. Emrick, Forming sticky droplets from slippery polymer zwitterions. *Adv. Mater.* **29**, 1702921 (2017).

39. O. Azzaroni, A. A. Brown, W. T. S. Huck, Ucst wetting transitions of polyzwitterionic brushes driven by self-association. *Angew. Chem. Int. Ed.* **45**, 1770–1774 (2006).

40. P. Mary, D. D. Bendejacq, M.-P. Labeau, P. Dupuis, Reconciling low- and high-salt solution behavior of sulfobetaine polyzwitterions. *J. Phys. Chem. B* **111**, 7767–7777 (2007).

41. M. Mukai, Y. Higaki, T. Hirai, A. Takahara, Separation of endo-cyclic 2-methacryloyloxyethyl choline phosphate by anion exchange approach. *Chem. Lett.* **47**, 1509–1511 (2018).

42. G. J. Hu, S. S. Parelkar, T. Emrick, A facile approach to hydrophilic, reverse zwitterionic, choline phosphate polymers. *Polym. Chem.* **6**, 525–530 (2015).

43. S. Mihara, K. Yamaguchi, M. Kobayashi, Intermolecular interaction of polymer brushes containing phosphorylcholine and inverse-phosphorylcholine. *Langmuir* **35**, 1172–1180 (2019).

44. Y. T. Zhang, Y. K. Yang, W. F. Ma, J. Guo, Y. Lin, C. C. Wang, Uniform magnetic core/shell microspheres functionalized with Ni^{2+}-iminodiacetic acid for one step purification and immobilization of His-tagged enzymes. *ACS Appl. Mater. Interfaces* **5**, 2626–2633 (2013).

45. C. Thery, K. W. Witwer, E. Aikawa, M. J. Alcaraz, J. D. Anderson, R. Andriantsitohaina, A. Antoniou, T. Arab, F. Archer, G. K. Atkin-Smith, D. C. Ayre, J. M. Bach, D. Bachurski, H. Baharvand, L. Balaj, S. Baldacchino, N. N. Bauer, A. A. Baxter, M. Bebawy, C. Beckham, A. B. Zavec, A. Benmoussa, A. C. Berardi, P. Bergese, E. Bielska, C. Blenkiron, S. Bobis-Wozowicz, E. Boilard, W. Boireau, A. Bongiovanni, F. E. Borras, S. Bosch, C. M. Boulanger, X. Breakefield, A. M. Breglio, M. A. Brennan, D. R. Brigstock, A. Brisson, M. L. D. Broekman, J. F. Bromberg, P. Bryl-Gorecka, S. Buch, A. H. Buck, D. Burger, S. Busatto, D. Buschmann, B. Bussolati, E. I. Buzas, J. B. Byrd, G. Camussi, D. R. F. Carter, S. Caruso, L. W. Chamley, Y. T. Chang, C. C. Chen, S. Chen, L. Cheng, A. R. Chin, A. Clayton, S. P. Clerici, A. Cocks, E. Cocucci, R. J. Coffey, A. Cordeiro-da-Silva, Y. Couch, F. A. W. Coumans, B. Coyle, R. Crescitelli, M. F. Criado, C. D'Souza-Schorey, S. Das, A. D. Chaudhuri, P. de Candia, E. F. De Santana, O. De Wever, H. A. del Portillo, T. Demaret, S. Deville, A. Devitt, B. Dhondt, D. Di Vizio, L. C. Dieterich, V. Dolo, A. P. D. Rubio, M. Dominici, M. R. Dourado, T. A. P. Driedonks, F. V. Duarte, H. M. Duncan, R. M.

Eichenberger, K. Ekstrom, S. E. L. Andaloussi, C. Elie-Caille, U. Erdbrugger, J. M. Falcon-Perez, F. Fatima, J. E. Fish, M. Flores-Bellver, A. Forsonits, A. Frelet-Barrand, F. Fricke, G. Fuhrmann, S. Gabrielsson, A. Gamez-Valero, C. Gardiner, K. Gartner, R. Gaudin, Y. S. Gho, B. Giebel, C. Gilbert, M. Gimona, I. Giusti, D. C. I. Goberdhan, A. Gorgens, S. M. Gorski, D. W. Greening, J. C. Gross, A. Gualerzi, G. N. Gupta, D. Gustafson, A. Handberg, R. A. Haraszti, P. Harrison, H. Hegyesi, A. Hendrix, A. F. Hill, F. H. Hochberg, K. F. Hoffmann, B. Holder, H. Holthofer, B. Hosseinkhani, G. K. Hu, Y. Y. Huang, V. Huber, S. Hunt, A. G. E. Ibrahim, T. Ikezu, J. M. Inal, M. Isin, A. Ivanova, H. K. Jackson, S. Jacobsen, S. M. Jay, M. Jayachandran, G. Jenster, L. Z. Jiang, S. M. Johnson, J. C. Jones, A. Jong, T. Jovanovic-Talisman, S. Jung, R. Kalluri, S. Kano, S. Kaur, Y. Kawamura, E. T. Keller, D. Khamari, E. Khomyakova, A. Khvorova, P. Kierulf, K. P. Kim, T. Kislinger, M. Klingeborn, D. J. Klinke, M. Kornek, M. M. Kosanovic, A. F. Kovacs, E. M. Kramer-Albers, S. Krasemann, M. Krause, I. V. Kurochkin, G. D. Kusuma, S. Kuypers, S. Laitinen, S. M. Langevin, L. R. Languino, J. Lannigan, C. Lasser, L. C. Laurent, G. Lavieu, E. Lazaro-Ibanez, S. Le Lay, M. S. Lee, Y. X. F. Lee, D. S. Lemos, M. Lenassi, A. Leszczynska, I. T. S. Li, K. Liao, S. F. Libregts, E. Ligeti, R. Lim, S. K. Lim, A. Line, K. Linnemannstons, A. Llorente, C. A. Lombard, M. J. Lorenowicz, A. M. Lorincz, J. Lotvall, J. Lovett, M. C. Lowry, X. Loyer, Q. Lu, B. Lukomska, T. R. Lunavat, S. L. N. Maas, H. Malhi, A. Marcilla, J. Mariani, J. Mariscal, E. S. Martens-Uzunova, L. Martin-Jaular, M. C. Martinez, V. R. Martins, M. Mathieu, S. Mathivanan, M. Maugeri, L. K. McGinnis, M. J. McVey, D. G. Meckes, K. L. Meehan, I. Mertens, V. R. Minciacchi, A. Moller, M. M. Jorgensen, A. Morales-Kastresana, J. Morhayim, F. Mullier, M. Muraca, L. Musante, V. Mussack, D. C. Muth, K. H. Myburgh, T. Najrana, M. Nawaz, I. Nazarenko, P. Nejsum, C. Neri, T. Neri, R. Nieuwland, L. Nimrichter, J. P. Nolan, E. N. M. Nolte-'t Hoen, N. N. Hooten, L. O'Driscoll, T. O'Grady, A. O'Loghlen, T. Ochiya, M. Olivier, A. Ortiz, L. A. Ortiz, X. Osteikoetxea, O. Ostegaard, M. Ostrowski, J. Park, D. M. Pegtel, H. Peinado, F. Perut, M. W. Pfaffl, D. G. Phinney, B. C. H. Pieters, R. C. Pink, D. S. Pisetsky, E. P. von Strandmann, I. Polakovicova, I. K. H. Poon, B. H. Powell, I. Prada, L. Pulliam, P. Quesenberry, A. Radeghieri, R. L. Raffai, S. Raimondo, J. Rak, M. I. Ramirez, G. Raposo, M. S. Rayyan, N. Regev-Rudzki, F. L. Ricklefs, P. D. Robbins, D. D. Roberts, S. C. Rodrigues, E. Rohde, S. Rome, K. M. A. Rouschop, A. Rughetti, A. E. Russell, P. Saa, S.

Sahoo, E. Salas-Huenuleo, C. Sanchez, J. A. Saugstad, M. J. Saul, R. M. Schiffelers, R. Schneider, T. H. Schoyen, A. Scott, E. Shahaj, S. Sharma, O. Shatnyeva, F. Shekari, G. V. Shelke, A. K. Shetty, K. Shiba, P. R. M. Siljander, A. M. Silva, A. Skowronek, O. L. Snyder, R. P. Soares, B. W. Sodar, C. Soekmadji, J. Sotillo, P. D. Stahl, W. Stoorvogel, S. L. Stott, E. F. Strasser, S. Swift, H. Tahara, M. Tewari, K. Timms, S. Tiwari, R. Tixeira, M. Tkach, W. S. Toh, R. Tomasini, A. C. Torrecilhas, J. P. Tosar, V. Toxavidis, L. Urbanelli, P. Vader, B. W. M. van Balkom, S. G. van der Grein, J. Van Deun, M. J. C. van Herwijnen, K. Van Keuren-Jensen, G. van Niel, M. E. van Royen, A. J. van Wijnen, M. H. Vasconcelos, I. J. Vechetti, T. D. Veit, L. J. Vella, E. Velot, F. J. Verweij, B. Vestad, J. L. Vinas, T. Visnovitz, K. V. Vukman, J. Wahlgren, D. C. Watson, M. H. M. Wauben, A. Weaver, J. P. Webber, V. Weber, A. M. Wehman, D. J. Weiss, J. A. Welsh, S. Wendt, A. M. Wheelock, Z. Wiener, L. Witte, J. Wolfram, A. Xagorari, P. Xander, J. Xu, X. M. Yan, M. Yanez-Mo, H. Yin, Y. Yuana, V. Zappulli, J. Zarubova, V. Zekas, J. Y. Zhang, Z. Z. Zhao, L. Zheng, A. R. Zheutlin, A. M. Zickler, P. Zimmermann, A. M. Zivkovic, D. Zocco, E. K. Zuba-Surma, Minimal information for studies of extracellular vesicles 2018 (MISEV2018): A position statement of the international society for extracellular vesicles and update of the MISEV2014 guidelines. *J. Extracell. Vesicles* **7**, 1535750 (2018).

46. R. E. Hewitt, A. McMarlin, D. Kleiner, R. Wersto, P. Martin, M. Tsoskas, G. W. H. Stamp, W. G. Stetler-Stevenson, Validation of a model of colon cancer progression. *J. Pathol.* **192**, 446–454 (2000).

47. R. Upadhya, L. N. Madhu, S. Attaluri, D. L. G. Gitai, M. R. Pinson, M. Kodali, G. Shetty, G. Zanirati, S. Kumar, B. Shuai, S. T. Weintraub, A. K. Shetty, Extracellular vesicles from human iPSC-derived neural stem cells: MiRNA and protein signatures, and anti-inflammatory and neurogenic properties. *J. Extracell. Vesicles* **9**, 1809064 (2020).

48. C. D. Walkey, W. C. W. Chan, Understanding and controlling the interaction of nanomaterials with proteins in a physiological environment. *Chem. Soc. Rev.* **41**, 2780–2799 (2012).

49. M. J. Huang, J. J. Yang, T. Wang, J. Song, J. L. Xia, L. L. Wu, W. Wang, Q. Y. Wu, Z. Zhu, Y. L. Song, C. Y. Yang, Homogeneous, low-volume, efficient, and sensitive quantitation of circulating exosomal PD-L1 for cancer diagnosis and immunotherapy response prediction. *Angew. Chem. Int. Ed.* **59**, 4800–4805 (2020).

50. J. N. Higginbotham, Q. Zhang, D. K. Jeppesen, A. M. Scott, H. C. Manning, J. Ochieng, J. L. Franklin, R. J. Coffey, Identification and characterization of EGF receptor in individual exosomes by fluorescence-activated vesicle sorting. *J. Extracell. Vesicles* **5**, 29254 (2016).

51. F. Tian, S. Zhang, C. Liu, Z. Han, Y. Liu, J. Deng, Y. Li, X. Wu, L. Cai, L. Qin, Q. Chen, Y. Yuan, Y. Liu, Y. Cong, B. Ding, Z. Jiang, J. Sun, Protein analysis of extracellular vesicles to monitor and predict therapeutic response in metastatic breast cancer. *Nat. Commun.* **12**, 2536 (2021).

52. A. Hoshino, H. S. Kim, L. Bojmar, K. E. Gyan, M. Cioffi, J. Hernandez, C. P. Zambirinis, G. Rodrigues, H. Molina, S. Heissel, M. T. Mark, L. Steiner, A. Benito-Martin, S. Lucotti, A. Di Giannatale, K. Offer, M. Nakajima, C. Williams, L. Nogues, F. A. P. Vatter, A. Hashimoto, A. E. Davies, D. Freitas, C. M. Kenific, Y. Ararso, W. Buehring, P. Lauritzen, Y. Ogitani, K. Sugiura, N. Takahashi, M. Aleckovic, K. A. Bailey, J. S. Jolissant, H. J. Wang, A. Harris, L. M. Schaeffer, G. Garcia-Santos, Z. Posner, V. P. Balachandran, Y. Khakoo, G. P. Raju, A. Scherz, I. Sagi, R. Scherz-Shouval, Y. Yarden, M. Oren, M. Malladi, M. Petriccione, K. C. De Braganca, M. Donzelli, C. Fischer, S. Vitolano, G. P. Wright, L. Ganshaw, M. Marrano, A. Ahmed, J. DeStefano, E. Danzer, M. H. A. Roehrl, N. J. Lacayo, T. C. Vincent, M. R. Weiser, M. S. Brady, P. A. Meyers, L. H. Wexler, S. R. Ambati, A. J. Chou, E. K. Slotkin, S. Modak, S. S. Roberts, E. M. Basu, D. Diolaiti, B. A. Krantz, F. Cardoso, A. L. Simpson, M. Berger, C. M. Rudin, D. M. Simeone, M. Jain, C. M. Ghajar, S. K. Batra, B. Stanger, J. Bui, K. A. Brown, V. K. Rajasekhar, J. H. Healey, M. de Sousa, K. Kramer, S. Sheth, J. Baisch, V. Pascual, T. E. Heaton, M. P. La Quaglia, D. J. Pisapia, R. Schwartz, H. Y. Zhang, Y. Liu, A. Shukla, L. Blavier, Y. A. DeClerck, M. LaBarge, M. J. Bissell, T. C. Caffrey, P. M. Grandgenett, M. A. Hollingsworth, J. Bromberg, B. Costa-Silva, H. Peinado, Y. B. Kang, B. A. Garcia, E. M. O'Reilly, D. Kelsen, T. M. Trippett, D. R. Jones, I. R. Matei, W. R. Jarnagin, D. Lyden, Extracellular

vesicle and particle biomarkers define multiple human cancers. *Cell* **182**, 1044–1061.e18 (2020).

53. S. J. Vermeulen, E. A. Bruyneel, M. E. Bracke, G. K. De Bruyne, K. M. Vennekens, K. L. Vleminckx, G. J. Berx, F. M. van Roy, M. M. Mareel, Transition from the noninvasive to the invasive phenotype and loss of alpha-catenin in human colon cancer cells. *Cancer Res.* **55**, 4722–4728 (1995).

54. K. O'Brien, K. Breyne, S. Ughetto, L. C. Laurent, X. O. Breakefield, RNA delivery by extracellular vesicles in mammalian cells and its applications. *Nat. Rev. Mol. Cell Biol.* **21**, 585–606 (2020).

55. K. Trajkovic, C. Hsu, S. Chiantia, L. Rajendran, D. Wenzel, F. Wieland, P. Schwille, B. Brugger, M. Simons, Ceramide triggers budding of exosome vesicles into multivesicular endosomes. *Science* **319**, 1244–1247 (2008).

56. J. Kowal, M. Tkach, C. Thery, Biogenesis and secretion of exosomes. *Curr. Opin. Cell Biol.* **29**, 116–125 (2014).

57. X. H. Liu, Z. Y. Zong, X. Z. Liu, Q. Li, A. Li, C. Xu, D. B. Liu, Stimuli-mediated specific isolation of exosomes from blood plasma for high-throughput profiling of cancer biomarkers. *Small Methods* **6**, 2101234 (2022).

58. X. Z. Liu, Z. Y. Zong, M. D. Xing, X. H. Liu, J. Li, D. B. Liu, pH-mediated clustering of exosomes: Breaking through the size limit of exosome analysis in conventional flow cytometry. *Nano Lett.* **21**, 8817–8823 (2021).

59. M. L. Merchant, I. M. Rood, J. K. J. Deegens, J. B. Klein, Isolation and characterization of urinary extracellular vesicles: Implications for biomarker discovery. *Nat. Rev. Nephrol.* **13**, 731–749 (2017).

60. R. Bandu, J. W. Oh, K. P. Kim, Mass spectrometry-based proteome profiling of extracellular vesicles and their roles in cancer biology. *Exp. Mol. Med.* **51**, 1–10 (2019).

61. I. K. Herrmann, M. J. A. Wood, G. Fuhrmann, Extracellular vesicles as a next-generation drug delivery platform. *Nat. Nanotechnol.* **16**, 748–759 (2021).

62. P. Escudé Martinez de Castilla, L. Tong, C. Huang, A. M. Sofias, G. Pastorin, X. Chen, G. Storm, R. M. Schiffelers, J.-W. Wang, Extracellular vesicles as a drug delivery system: A systematic review of preclinical studies. *Adv. Drug Deliv. Rev.* **175**, 113801 (2021).

63. K. W. Witwer, B. W. M. Van Balkom, S. Bruno, A. Choo, M. Dominici, M. Gimona, A. F. Hill, D. De Kleijn, M. Koh, R. C. Lai, S. A. Mitsialis, L. A. Ortiz, E. Rohde, T. Asada, W. S. Toh, D. J. Weiss, L. Zheng, B. Giebel, S. K. Lim, Defining mesenchymal stromal cell (MSC)-derived small extracellular vesicles for therapeutic applications. *J. Extracell. Vesicles* **8**, 1609206 (2019).

64. L. M. Doyle, M. Z. Wang, Overview of extracellular vesicles, their origin, composition, purpose, and methods for exosome isolation and analysis. *Cell* **8**, 727 (2019).

65. T. Hu, J. Wolfram, S. Srivastava, Extracellular vesicles in cancer detection: Hopes and hypes. *Trends Cancer* **7**, 122–133 (2021).
